# PLK1 regulates the PrimPol damage tolerance pathway during the cell cycle

**DOI:** 10.1126/sciadv.abh1004

**Published:** 2021-12-03

**Authors:** Laura J. Bailey, Rebecca Teague, Peter Kolesar, Lewis J. Bainbridge, Howard D. Lindsay, Aidan J. Doherty

**Affiliations:** 1 Genome Damage and Stability Centre, University of Sussex, Falmer, Brighton BN1 9RQ, UK.; 2 Lancaster Medical School, Faculty of Health and Medicine, Lancaster University, Lancaster LA1 4YQ, UK.

## Abstract

Replication stress and DNA damage stall replication forks and impede genome synthesis. During S phase, damage tolerance pathways allow lesion bypass to ensure efficient genome duplication. One such pathway is repriming, mediated by Primase-Polymerase (PrimPol) in human cells. However, the mechanisms by which PrimPol is regulated are poorly understood. Here, we demonstrate that PrimPol is phosphorylated by Polo-like kinase 1 (PLK1) at a conserved residue between PrimPol’s RPA binding motifs. This phosphorylation is differentially modified throughout the cell cycle, which prevents aberrant recruitment of PrimPol to chromatin. Phosphorylation can also be delayed and reversed in response to replication stress. The absence of PLK1-dependent regulation of PrimPol induces phenotypes including chromosome breaks, micronuclei, and decreased survival after treatment with camptothecin, olaparib, and UV-C. Together, these findings establish that deregulated repriming leads to genomic instability, highlighting the importance of regulating this damage tolerance pathway following fork stalling and throughout the cell cycle.

## Teaser

PrimPol is regulated by PLK1 phosphorylation to restrict repriming and prevent genomic instability.

## INTRODUCTION

The DNA replication machinery regularly encounters obstacles that slow or stall its progression. The causes of replicase stalling are varied and include DNA lesions and structures, nucleotide depletion, and other forms of genotoxic stress (*1*). To complete replication, cells must bypass such impediments, as unresolved forks are susceptible to degradation and may induce double-strand breaks. Cells have evolved several DNA damage tolerance (DDT) pathways to maintain ongoing replication under perturbed conditions, whose usage is dependent on the environment, type of blockage, and available resources.

One such DDT pathway involves repriming DNA synthesis downstream of obstacles to enable stalled replication to resume. Repriming in human cells is dependent on Primase-Polymerase (PrimPol), an enzyme involved in the maintenance of nuclear and mitochondrial DNA replication (*2*, *3*). PrimPol can reprime DNA synthesis on the leading strand after the fork encounters stalling lesions, such as cyclopyrimidine dimers and structured DNA (e.g., G4 quadruplexes), as well as chain terminators (*4*–*7*). PrimPol can also perform translesion synthesis (TLS) polymerase, replicating across DNA lesions (e.g., 8-oxoG and 6-4 photoproducts) that stall replicative polymerases (*6*, *7*).

Specialized DNA polymerases, such as Pol Eta (η), can also perform TLS (*8*, *9*). This allows for continuous replication and lesion bypass at the expense of fidelity (*10*). Other DDT pathways, involving fork reversal, provide more error-free bypass mechanisms (*11*). As many DDT pathways are available during DNA replication, it is essential that cells use the optimal restart pathway to efficiently reinitiate genome synthesis.

The availability of alternative overlapping DDT pathways means that, despite its importance in maintaining active replication forks, cells lacking PrimPol are viable and grow normally. However, these cells have delayed recovery times after ultraviolet C (UV-C) damage and hallmarks of replication stress, such as increased micronuclei and elevated mutation frequencies (*6*, *12*, *13*). These phenotypes are further exacerbated when other DDT pathways are disrupted, such as in the absence of Pol η, where PrimPol-null cells exhibit an overt UV-C sensitivity (*4*, *6*, *12*). Recent studies have also shown that in the absence of HLTF, an enzyme involved in fork reversal, PrimPol-mediated repriming or TLS is used to rescue stalled forks (*14*). Similarly, in the absence of CARM1/PRMT4, implicated in the stabilization of reversed forks, PrimPol and TLS are both used to restart replication forks (*15*). PrimPol also plays a role in DDT when cells lack BRCA1, with PrimPol protein levels increasing after multiple cisplatin doses, leading to suppressed fork reversal (*16*).

While PrimPol can operate when other pathways are unavailable, it is unknown how PrimPol is prevented from acting when repriming is undesirable. PrimPol must be tightly regulated during DNA replication to avoid aberrant repriming, fork speeding, and chromosomal breaks (*15*), and such regulation would therefore need to be dynamic to respond to DNA damage or changes in DDT pathway availability. While recent studies have shown that PrimPol protein levels are tightly controlled by ATR activity and regulated by WRNIP1 levels (*16*–*18*), there is currently no evidence for a more responsive mode of regulation.

Posttranslational modifications (PTMs) provide a dynamic and reversible form of regulation that is known to play roles in regulating DDT pathways. TLS polymerases, such as Pol η, are highly regulated by PTMs, such as phosphorylation, ubiquitination, and SUMOylation (*19*–*22*). To control phosphorylation, cells use a number of specific kinases and phosphatases to regulate progression through the cell cycle and the cell’s response to replication stress. One such kinase is Polo-like kinase 1 (PLK1), which has a critical role in mitotic progression and has also been implicated in the DNA damage response (*23*–*25*). PLK1 regulates many DNA damage response proteins, including Rad51, BRCA2, and MRE11 (*26*–*28*). PLK1 itself is also regulated by a defined methylation/phosphorylation switch, important for the timely removal of RPA2 (Replication Protein A2) and Rad51 from DNA damage sites (*29*).

Here, we report that PrimPol is phosphorylated by PLK1 on a highly conserved serine residue, located between two RPA binding sites at its C terminus. Phosphorylation by PLK1 occurs at the end of S phase, although it can be modulated in response to high levels of replication stress, such as that induced by the poly(ADP-ribose) polymerase (PARP) inhibitor olaparib or the topoisomerase poison camptothecin. Deregulation of this PLK1-dependent phosphorylation leads to damage sensitivity and the onset of cellular phenotypes associated with genomic instability, including micronuclei, chromosome breaks, and mitotic defects. Together, these findings establish that PLK1 provides regulatory control over PrimPol during the cell cycle to restrict its activity outside of S phase, where it is required for restarting stalled forks to ensure that DNA replication proceeds in a timely and efficient manner.

## RESULTS

### PrimPol is phosphorylated at a conserved serine located between the RPA binding motifs

**Fig. 1.**
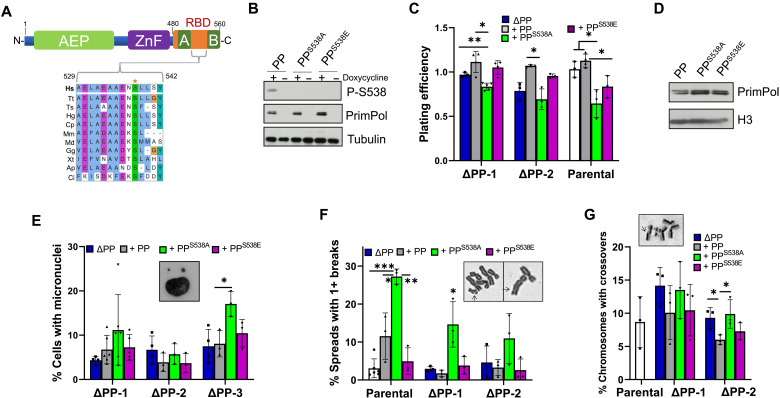
PrimPol is modified by phosphorylation at S538, and loss of this causes genomic instability. (**A**) Alignment of the C-terminal region of PrimPol (PP) containing the RPA binding domain (RBD). Hs, *Homo sapien* (human); Tt, *Tursiops truncatus* (Atlantic bottle-nosed dolphin); Ts, *Tarsius syrichta* (Philippine tarsier); Hg, *Heterocephalus glaber* (naked mole rat); Cp, *Cavia porcellus* (guinea pig); Mm, *Mus musculus* (mouse); Md, *Monodelphis domestica* (gray short-tailed opossum); Gg, *Gallus gallus* (chicken); Xt, *Xenopus tropicalis* (Western clawed frog); Ap, *Anas platyrhynchos* (Northern mallard); Ci, *Ciona intestinalis* (vase tunicate); AEP, Archaeao-Eukaryotic Primase. (**B**) Protein phosphorylation was examined in whole-cell lysate from HEK293 cells where PrimPol was expressed by addition of doxycycline for 24 hours, analyzed by Western blotting. (**C**) Plating efficiency of different cell lines expressing PrimPol variants compared to no-doxycycline controls. (**D**) ΔPP-1 cells expressing PrimPol, PrimPol^S538A^, or PrimPol^S538E^ were collected, and detergent-resistant chromatin fractions were separated from soluble proteins before being separated by Western blot. (**E**) Quantification of cells with one or more micronuclei 48 hours after PrimPol expression. (**F**) PrimPol was expressed for 96 hours in HEK293 cells before being stalled in mitosis with nocodazole. Chromosomes were spread, and those containing one or more chromosome with a break were quantified as a percentage of the population. (**G**) To analyze sister chromatid exchanges, cells were grown in 10 μM BrdU for 48 hours before being blocked in mitosis. Cells were spread and stained with Hoescht and Geimsa, and the number of chromosomes with one or more crossovers was counted as a percentage of the total population.

Despite recent progress, little is known about how PrimPol’s activity and recruitment are regulated throughout the cell cycle. Given that its interaction with RPA is requisite for PrimPol’s functionality at stalled forks, PTMs in the C terminus may play a role in its regulation. We carried out mass spectrometry analysis on Flag-tagged PrimPol from human cells to detect potential modifications that may play roles in regulating its activity. We identified a number of phosphorylation sites (fig. S1A), including a serine residue (S538) located between the two RPA binding motifs (RBMs) in human PrimPol. We also identified phosphorylation of a homologous residue on the C terminus of *Xenopus laevis* PrimPol and found that this modification is conserved with the duplication of the RBM B (fig. S1A). As this residue is also highly conserved in other eukaryotes (Fig. 1A), we hypothesized that it may play a role in regulating PrimPol. To study the significance of this phosphorylation in human cells, we generated a phospho-specific antibody, raised against a peptide containing phosphorylated S538 (P-S538). To confirm this antibody’s specificity, we tested it against whole-cell extracts from human embryonic kidney (HEK) 293 cells expressing PrimPol or mutants PrimPol^S538A^ and PrimPol^S538E^ (Fig. 1B). While S538 phosphorylation was observed for wild-type (WT) protein, no binding was observed when S538 was mutated. In addition, antibody binding was also lost when cell lysate was treated with λ phosphatase (fig. S1B).

### Disruption of S538 phosphorylation affects cell growth and genome stability

To analyze the significance of S538 phosphorylation, we examined the effects that disrupting this modification had in cultured human cells. We first generated several PrimPol^−/−^ clones in a Flp-In HEK293 T-REx cell line, denoted ΔPP-1-3. These cells all carried biallelic *PrimPol* deletions, which lead to downstream gene disruption, with no observed PrimPol protein production (fig. S1C). ΔPP cells exhibited phenotypes similar to those observed previously in MRC5 PrimPol^−/−^ cells, namely, normal growth and no UV-C sensitivity, but delayed cell cycle recovery after UV damage (fig. S1, D to G) (*12*). N-terminal FLAG-tagged PrimPol or PrimPol containing S538 mutations was stably introduced into the Flp-In T-REx site in these cells and expressed using doxycycline (Fig. 1B and fig. S2A) (*30*). To analyze any defect arising from the expression of mutant PrimPol, we used colony formation assays in the presence or absence of doxycycline to assess plating efficiency. While PrimPol and PrimPol^S538E^ expression had no effect on plating efficiency, PrimPol^S538A^ caused a significant decrease in colony formation (Fig. 1C). In addition, while expression of PrimPol had little effect on growth rates, PrimPol^S538A^ caused a small but significant increase in doubling times, suggesting that this modification may play a role in PrimPol’s ability to maintain cell cycle progression or cell viability (fig. S2B). We also observed a similar effect on plating efficiency when we expressed PrimPol^S538A^ in parental cells carrying endogenous PrimPol protein, indicating that this mutation has a dominant negative effect (Fig. 1C). However, we found no significant changes in cell cycle populations 48 hours after protein induction (fig. S2C).

As S538 resides between the RBMs, it may regulate PrimPol’s interaction with RPA and thus its recruitment and retention on single-stranded DNA (ssDNA). However, when we analyzed the binding of the C-terminal region of WT or mutant PrimPol with RPA70N by analytical gel filtration, we observed no overt changes in the interactions between PrimPol and RPA in vitro (fig. S3A). We also found no changes in PrimPol and RPA interaction in vivo after mutation of the S538 residue when analyzed by immunoprecipitation (fig. S3B). In addition, mutation of S538 did not affect PrimPol’s chromatin association in vivo (Fig. 1D). Notably, biochemical analysis also showed that neither PrimPol^S538A^ nor PrimPol^S538E^ altered primase or polymerase activities or fidelity in vitro (fig. S3, C and D).

To understand the impact of mutating the S538 phosphorylation site, we examined markers of genomic instability. Cells expressing PrimPol^S538A^ showed an increase in micronuclei under unperturbed conditions (Fig. 1E) and substantially more chromosomal breaks (Fig. 1F). As with PrimPol^−/−^ MRC5 cells, ΔPP cells exhibited increased sister chromatid exchanges (SCEs) that could be rescued by expression of PrimPol or PrimPol^S538E^ but not PrimPol^S538A^ (Fig. 1G) (*12*). Together, these results indicate that PrimPol^S538A^ expression leads to an increase in genomic instability.

### PrimPol^S538A^ decreases cell viability after UV-C–induced DNA damage

**Fig. 2.**
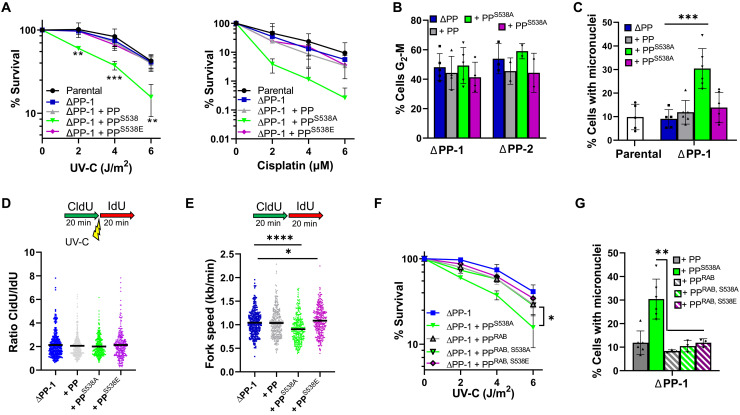
Loss of PrimPol S538 phosphorylation affects genomic stability after UV-C damage and is dependent on RPA interaction. (**A**) Damage sensitivity was measured by colony survival after increasing doses of UV-C (left) or cisplatin (right). (**B**) Quantification of cell cycle recovery after damage was measured by flow cytometry, using EdU and PI labeling 24 hours after treatment with 5 J/m^2^ UV-C, images shown in fig. S4B. (**C**) Cells with one or more micronuclei were counted 48 hours after 5 J/m^2^ UV-C treatment. (**D**) CldU/IdU ratios show replication changes after a pulse of 20 J/m^2^ UV-C was given between labels. (**E**) Undamaged replication fork speeds were measured in the different cell lines at least 16 hours after PrimPol expression by labeling cells consecutively with CldU and IdU. (**F**) UV sensitivity was analyzed by colony survival in ΔPP-1 cells expressing RAB mutated forms of PrimPol also carrying the 538 mutations. (**G**) The effect of loss of PrimPol’s RPA interaction in micronuclei formation was compared in cells expressing PrimPol mutants additionally carrying the RAB mutations, 48 hours after exposure to 5 J/m^2^ UV-C.

As PrimPol is required for repriming DNA replication after fork stalling lesions and structures, we examined the role of S538 phosphorylation in maintaining DNA replication and cell viability after DNA damage (*4*, *5*, *7*). After treatment with UV-C, we observed a significant increase in damage sensitivity in cells expressing the phospho-null mutant (PrimPol^S538A^) compared to ΔPP cells (Fig. 2A), establishing that expressing PrimPol^S538A^ is more harmful than the absence of PrimPol. To confirm that this was not an artifact of an individual clone, we carried out UV-C survival assays on three independent PrimPol knockout clones complemented with PrimPol^S538A^ and obtained consistent results (fig. S4A). In addition, we observed a decrease in UV-C survival when expressing PrimPol^S538A^ in parental cells, which also have endogenous protein, indicating that endogenous levels of PrimPol are not able to overcome the toxicity of PrimPol^S538A^ (fig. S4A). Moreover, cells expressing PrimPol^S538A^ also showed a sensitivity to the cross-linking agent cisplatin (Fig. 2A and fig. S4A). As previously reported, PrimPol^−/−^ cells exhibit delayed recovery after UV-C (*12*); we therefore measured the ability of the different PrimPol proteins to complement this defect. As in MRC5 PrimPol^−/−^ cells, we observed that expression of PrimPol and PrimPol^S538E^ decreased the delay in recovery time 24 hours after UV-C damage. While cells expressing PrimPol^S538A^ showed less delay than ΔPP cells, there was an increase in late S-G_2_-M stalling (Fig. 2B and fig. S4B). We also examined hallmarks of genome instability and found a significant increase in micronuclei after UV-C damage in cells expressing PrimPol^S538A^, compared to those expressing PrimPol or PrimPol^S538E^ (Fig. 2C and fig. S4C). To confirm that PrimPol^S538A^ toxicity was not specific to HEK293 cells, we expressed PrimPol in RPE1 cells using a Sleeping Beauty transposon expression system (fig. S4D) (*31*). We found that PrimPol^S538A^ expression caused a similar decrease in survival after UV-C damage, along with hallmarks of genomic instability, such as increased micronuclei both with and without UV-C damage (fig. S4, E and F).

In asynchronous cells damaged with UV-C (6 J/m^2^), there was no detectable difference in the amount of mutant PrimPol bound to chromatin, compared to PrimPol (fig. S5A). To look more directly at the effects of PrimPol^S538A^ at the replication fork, we used a DNA fiber assay to analyze ongoing replication after UV damage. Despite the increased UV-C sensitivity, both PrimPol^S538A^ and PrimPol^S538E^ had minimal effect on fork stalling (Fig. 2D) This suggests that the genotoxic effects of PrimPol^S538A^ may be initiated outside of S phase replication.

In addition, we found that PrimPol^S538A^ expression caused a decrease in replication fork speed under undamaged conditions (Fig. 2E and fig. S5B). We observed a small but significant increase in replication fork speeds with PrimPol^S538E^ expression, suggesting that this mutant is also capable of causing changes in fork progression, potentially through alteration of pathway choice at stalled or slowed forks (Fig. 2E). This was also observed in parental cells containing endogenous PrimPol (fig. S5B). To examine whether PrimPol’s activity was inhibited after loss of phosphorylation, we looked at the restart of stalled forks after HU or camptothecin treatment and identified a decrease in fork restart in the absence of PrimPol. However, mutation of S538 did not affect the ability of the protein to complement this, confirming that, as observed in vitro, the protein remains functional in the absence of S538 phosphorylation (fig. S5C).

### PrimPol^S538A^-induced genotoxicity is rescued by mutation of RBMs

To confirm whether PrimPol^S538A^’s phenotype was due directly to its activity on DNA, we investigated whether impairing PrimPol’s recruitment to chromatin, by mutation of PrimPol’s RBMs, could abolish these phenotypes. RBM-A and RBM-B, the two motifs responsible for RPA association and therefore recruitment to ssDNA, were mutated to generate PrimPol^RAB^ in the presence or absence of the S538 mutations (fig. S5D) (*30*). When expressed alone in ΔPP cells, PrimPol^RAB^ had little effect on UV-C survival and rescued PrimPol^S538A^’s UV sensitivity (Fig. 2F and fig. S5E). In addition, complete loss of both RPA binding sites (PrimPol^RAB^) completely reversed the genomic instability phenotypes observed with PrimPol^S538A^, evident by no increase in chromosome breaks or micronuclei (Fig. 2G and fig. S5F). Together, these findings establish that the genotoxicity induced by PrimPol^S538A^ requires PrimPol’s RPA-dependent interaction with chromatin.

### PrimPol^S538A^ causes sensitivity to olaparib and camptothecin

**Fig. 3.**
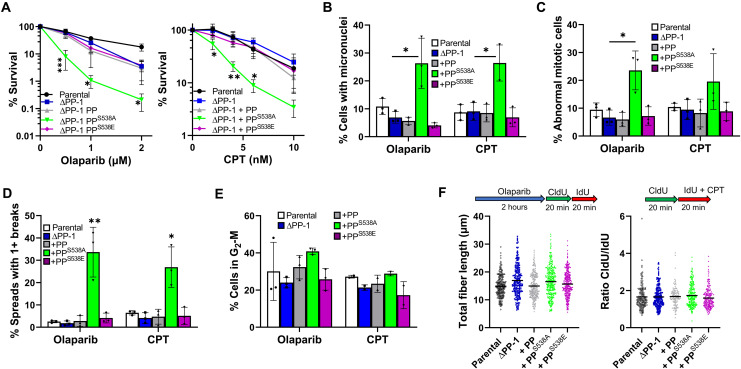
Cells expressing PrimPol^S538A^ **are sensitive to genotoxic agents camptothecin and olaparib.** (**A**) Sensitivity to olaparib (left) or camptothecin (CPT) (right) was measured by colony survival approximately 10 days after the addition of drugs. Changes in levels of micronuclei (**B**) or cells undergoing abnormal mitotic segregation (**C**) were analyzed 48 hours after treatment with camptothecin or olaparib. (**D**) Chromosome breaks were analyzed 48 hours after treatment with olaparib or camptothecin. (**E**) Effects of olaparib and camptothecin were quantified after 48 hours by flow cytometry; see images in fig. S6F. (**F**) Effects of olaparib and camptothecin on replication speeds were analyzed by measuring DNA fibers. Cells were treated with 10 μM olaparib for 2 hours before and during fiber analysis, or 50 nM camptothecin was added with IdU labeling.

Recent studies have shown that repriming and fork reversal are distinct DDT mechanisms that rescue stalled replication forks. Changes in the levels of fork reversal proteins, such as HLTF and PARP, have been suggested to alter the balance between these pathways and therefore the requirement for PrimPol following stalling (*14*, *15*). Therefore, we used the drug olaparib to inhibit PARP and determine whether the S538A mutation affected the availability of PrimPol for repriming at stalled forks. When we examined colony formation in the presence of low doses of olaparib, we observed that expression of PrimPol^S538A^ caused a significant increase in sensitivity, similar to that observed with other damaging agents (Fig. 3A and fig. S6A). However, in addition to affecting the availability of the fork reversal pathway, PARP is also used in the resolution of single-stranded and double-stranded breaks (SSBs and DSBs); it therefore follows that the use of olaparib leads to an increase in SSBs and DSBs (*32*, *33*). We investigated this possibility by using the Top1 poison camptothecin, which has been shown to cause an increase in SSBs (*34*). We observed that, while little sensitivity was evident in cells lacking PrimPol, cells expressing PrimPol^S538A^ showed a significant decrease in cell survival after the addition of camptothecin (Fig. 3A and fig. S6B). These combined sensitivities suggest that this is due largely to an increase in breaks and fork stalling itself rather than the loss of fork reversal, as this is not affected by camptothecin treatment.

We then inspected for other signs of genomic instability and stress after treatment with olaparib or camptothecin and again found an increase in micronuclei 48 hours after treatment only in cells expressing PrimPol^S538A^ (Fig. 3B and fig. S6C). We also observed a similar increase in mitotic cells showing abnormalities, such as lagging or misaligned chromosomes (Fig. 3C and fig. S6D). In addition, we noted a significant increase in chromosomes with breaks in cells expressing PrimPol^S538A^, 48 hours after treatment with camptothecin or olaparib (Fig. 3D and fig. S6E).

A small change in cell cycle population was observed after olaparib treatment, with cells expressing PrimPol^S538A^ stalling in G_2_-M more compared to cells expressing PrimPol or PrimPol^S538E^ (Fig. 3E and fig. S6F). As olaparib treatment causes an increase in replication speed (*35*), we investigated whether PrimPol may be involved in this process. While fork speeds increased after olaparib treatment, only minor differences were observed in cells expressing PrimPol^S538A^, suggesting that S538 phosphorylation does not play a significant role in this increase in replication fork speed (Fig. 3F). This is similar to other studies, where no changes in fiber length were observed in the absence of PrimPol after olaparib treatment (*15*). Camptothecin is known to cause fork stalling due to DNA torsional restraint (*36*). However, although we observed an increase in CldU (5-chloro-2′-deoxyuridine)/IdU (5-Iodo-2′-deoxyuridine) after addition of 50 nM camptothecin with the second label compared to untreated cells, no additional sensitivity was observed in cells expressing PrimPol^S538A^ (Fig. 3F). Again, this confirms that changes in S538 phosphorylation do not significantly affect its role during replication perturbation in S phase and support an effect in G_2_-M.

### Mutation of the Zn finger significantly rescues cellular defects caused by PrimPol^S538A^

**Fig. 4.**
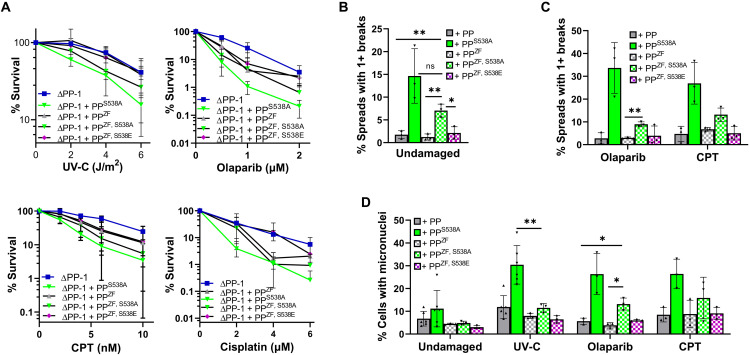
Mutation of PrimPol’s ZF reduces PrimPol^S538A^ **-induced genomic instability.** (**A**) Damage sensitivity changes caused by the addition of the ZF mutations in PrimPol were measured by colony survival with increasing doses of UV-C, cisplatin, olaparib, and camptothecin. Cells with one or more chromosome breaks were counted 96 hours after PrimPol expression (**B**) or after 48 hours of incubation with olaparib or camptothecin (**C**). (**D**) Micronuclei were counted 48 hours after recovery from 0 or 5 J/m^2^ UV-C or 48 hours of incubation with olaparib or camptothecin. ns, not significant.

As our data suggest that the S538A mutation may only have very minor effects during unperturbed S phase, we next examined whether the phenotypes induced by expression of PrimPol^S538A^ were dependent on PrimPol’s primase activity. PrimPol’s zinc finger (ZF) is required for its primase function and may be important for stabilization of the incoming nucleotide, primer translocation, and extension (*7*, *37*). Therefore, to further investigate the cause of PrimPol^S538A^-induced cell toxicity, we generated a disruptive ZF mutant (C419A, H426A, hereafter PrimPol^ZF^) in the PrimPol^S538A^ background (fig. S7A). Expression of PrimPol^ZF^ alone did not alter plating efficiency, but mutation of the ZF rescued the plating deficiency observed in PrimPol^S538A^ (fig. S7B). Expression of PrimPol^ZF^ also caused a small decrease in survival after treatment with UV-C, olaparib, camptothecin, or cisplatin, suggesting that its primase activity is required for restart after such damage (Fig. 4A). The combined PrimPol^ZF, S538A^ mutant was able to partially rescue the S538A-induced decrease in survival after damage but was still less viable than ΔPP or PrimPol^ZF^-expressing cells. In addition, PrimPol^ZF^ was able to largely rescue the increase in chromosomal breaks, micronuclei, and abnormal mitotic cells observed after expression of PrimPol^S538A^ in both damaged and undamaged cells (Fig. 4, B to D, and fig. S7, C to E). However, although levels of micronuclei and breaks were significantly lower, they were still consistently higher in PrimPol^ZF, S538A^-expressing lines compared with those expressing PrimPol. Although substantial loss of genomic instability was observed after the addition of PrimPol^ZF^, we noted that PrimPol, PrimPol^ZF^, and PrimPol^ZF S538A^ all proficiently bound chromatin in undamaged cells and cells damaged by UV-C, suggesting that recruitment is maintained (fig. S7F).

### S538 is phosphorylated by PLK1 in human cells

**Fig. 5.**
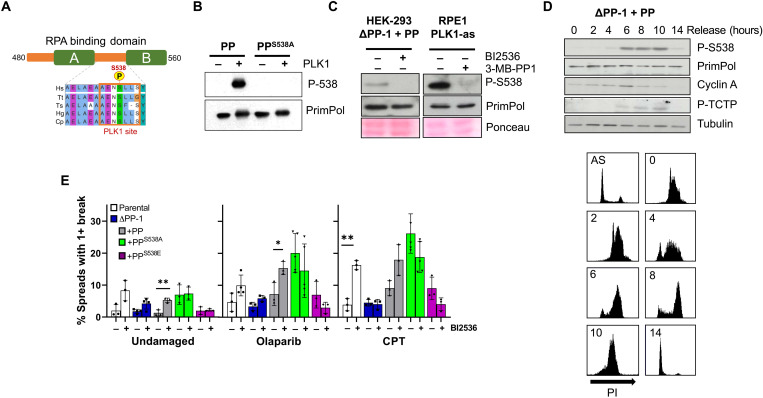
PrimPol S538 phosphorylation is cell cycle regulated by PLK1. (**A**) Alignment of the region of PrimPol containing a predicted PLK1 site (orange box) in different species, residue 538 in human protein. Hs, *H. sapien* (human); Tt, *T. truncatus* (Atlantic bottle-nosed dolphin); Ts, *T. syrichta* (Philippine tarsier); Hg, *H. glaber* (naked mole rat); Cp, *C. porcellus* (guinea pig). (**B**) In vitro PLK1 kinase assays where PrimPol or PrimPol^S538A^ was incubated with recombinant PLK1 and the resulting protein phosphorylation analyzed by Western blotting. (**C**) HEK293 ΔPP-1 cells expressing PrimPol were treated with BI2536 or mock treated (left). RPE1 PLK1-as cells expressing PrimPol were treated with 3-MB-PP1, and whole-cell lysate was subjected to Western blotting (right). (**D**) HEK293 ΔPP-1 cells expressing PrimPol were released from a double thymidine block for increasing times or left untreated as an asynchronous control and analyzed for cell cycle synchronization by flow cytometry (bottom). Whole-cell extract from cells at each time point was subjected to Western blotting (top). (**E**) Chromosome breaks were analyzed after synchronization, and cells were released from a double thymidine block into nocodazole with or without the addition of PLK1 inhibitor BI2536, olaparib, or camptothecin and allowed to progress to mitosis.

As ablation of PrimPol S538 phosphorylation clearly has significant effects on cellular viability, it appears likely that this modification is tightly regulated. To identify the kinase responsible for this phosphorylation, we analyzed the sequence motifs around S538 using the Eukaryotic Linear Motif database (*38*) and identified that it resides within a signature motif characteristic of a PLK1 site (*39*, *40*). This PLK1 motif is also highly conserved in PrimPol across a diverse range of higher eukaryotes (Fig. 5A). To determine whether the proposed motif containing S538 represents a bona fide PLK1 phosphorylation site, we purified recombinant PrimPol and performed kinase assays by incubating it with PLK1 and adenosine 5′-triphosphate (ATP). Using the P-S538–specific antibody, we showed that PrimPol, but not PrimPol^S538A^, was specifically phosphorylated by PLK1 at residue S538 in vitro (Fig. 5B).

To confirm whether this residue was also modified by PLK1 in vivo, we used the PLK-specific inhibitor BI2536 (*41*). Addition of BI2536 resulted in an absence of S538 phosphorylation, as identified using the phospho-specific antibody (Fig. 5C). To verify that S538 is specifically phosphorylated by PLK1 in cells, we used RPE1 PLK1-as cells (*42*). These cells contain a mutant form of PLK1, which can be inactivated using an ATP analog. Cells were stably transfected to express PrimPol before treatment with the ATP analog 3-MBPP1 to inactivate PLK1. In the absence of active PLK1, S538 phosphorylation was not detected, establishing a specific role for PLK1 kinase in phosphorylating PrimPol (Fig. 5C).

### Phosphorylation of PrimPol S538 is cell cycle regulated

PLK1 activity changes markedly throughout the cell cycle (*43*–*45*). Therefore, we examined PrimPol’s S538 phosphorylation levels across the cell cycle using thymidine synchronization. Phosphorylation of S538 was lost in cells synchronized at the G_1_-S border. Phosphorylation was then detectable by late S phase, reaching a peak in G_2_ (Fig. 5D). This phosphorylation was retained throughout mitosis before decreasing in G_1_. The same distribution of S538 phosphorylation was also observed in RPE1 cells across the cell cycle (fig. S8A). This pattern of phosphorylation matched that of known PLK1 substrates, such as TCTP (Fig. 5D) (*46*). This establishes that S538 phosphorylation is tightly regulated across the cell cycle, which suggests that it may be used to regulate PrimPol during different cell cycle stages.

To confirm the importance of PLK1 phosphorylation in the regulation of PrimPol activity in vivo, we again used the PLK1 inhibitor BI2536 (*41*). Cells were synchronized using a thymidine block, stalling cells at the G_1_-S border. Cells were then released into media containing nocodazole, and additionally containing olaparib or camptothecin, in the presence or absence of BI2536. Mitotic cells were collected and spread to analyze the accumulation of chromosome breaks. As observed previously, cells expressing PrimPol^S538A^ had an increased number of breaks under all conditions compared to ΔPP cells or those expressing PrimPol (Fig. 5E and fig. S8B). The addition of the PLK1 inhibitor did not further increase chromosomal breaks in PrimPol^S538A^ cells, nor did it increase the number of breaks in ΔPP cells, suggesting that the inhibition of PLK1 alone did not induce breaks. However, breaks increased in cells expressing PrimPol to levels similar to PrimPol^S538A^ cells. These findings suggest that preventing active phosphorylation of PrimPol by PLK1 is able to phenocopy the genotoxicity caused by the S538A mutation.

### PrimPol^S538A^ phenotypes are caused by its dysregulation across the cell cycle

**Fig. 6.**
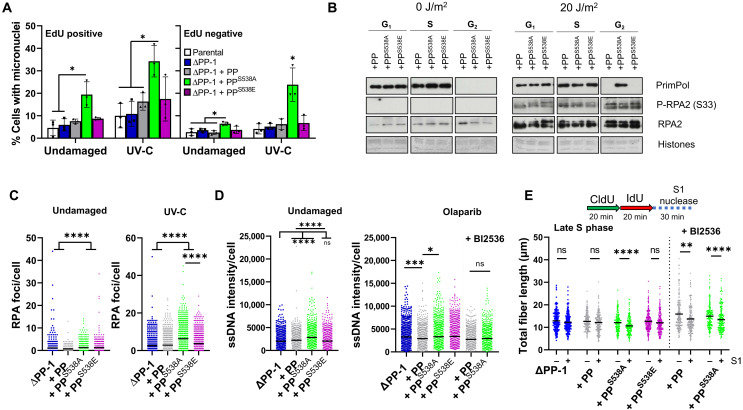
Dysregulation of PrimPol across the cell cycle drives genotoxic phenotypes. (**A**) To quantify micronuclei in cells, they were first labeled with EdU to distinguish those in S phase. Cells were treated with 0 or 5 J/m^2^ UV-C and allowed to recover for 48 hours before being analyzed for micronuclei and EdU. (**B**) Cells were synchronized by double thymidine block and released into respective cell cycle stages before being UV-C damaged and allowed to recover for 1 hour. Chromatin fractions were then isolated and analyzed by Western blotting. (**C**) RPA2 foci were quantified in undamaged cells or 24 hours after UV-C treatment, representative images shown in fig. S9D. (**D**) Cells were labeled with CldU for 48 hours before being washed and left untreated or treated with 10 μM olaparib for 2 hours. Cells were then stained for CldU under native conditions to analyze ssDNA. Images were quantified in ImageJ, *n* = 3 (olaparib, *N* = 2), examples shown in fig. S9F. (**E**) ssDNA gaps in newly replicated DNA, specifically in late S phase, were measured using the S1 fiber assay on cells synchronized with a double thymidine block. Cells were released for 6 hours with or without BI2536 and labeled with CldU and IdU before S1 nuclease treatment, and total fiber length was measured to assess nuclease cutting, *n* = 2.

As S538 phosphorylation is largely restricted to G_2_ and mitosis, PrimPol^S538A^ expressed in these cell cycle stages may be a potential cause of genotoxicity. To investigate changes in genomic stability across different cell cycle stages, we examined the formation of micronuclei. Cells were first labeled with EdU (5-ethynyl-2′-deoxyuridine) to identify those in S phase and then immediately treated with 0 or 5 J/m^2^ UV-C and allowed to recover for up to 48 hours. Cells expressing PrimPol^S538A^ had a significantly higher percentage of EdU-positive cells with micronuclei, 48 hours after labeling, suggesting that they may be retained by the cell for longer. When UV-C–treated cells were analyzed, we observed that in ΔPP-1 cells and those expressing PrimPol or PrimPol^S538E^, most micronuclei were found in cells that were in S phase when they were damaged (Fig. 6A and fig. S9A). EdU-negative cells, cells damaged by UV-C outside of S phase, showed little increase in micronuclei, even 48 hours after UV-C damage. This confirms that damage in nonreplicating cells is not a major cause of micronuclei in ΔPP-1 cells or those expressing PrimPol or PrimPol^S538E^. In contrast, cells expressing PrimPol^S538A^ showed a significant increase in micronuclei, in both EdU-positive and EdU-negative cells. These data show that expression of PrimPol^S538A^ after UV-C damage leads to micronuclei, regardless of whether the damage occurred in replicating or nonreplicating cells.

To examine changes in the recruitment of PrimPol^S538A^ outside of S phase, we analyzed cell stage–specific chromatin binding. Cells were first synchronized using a double thymidine block and released to progress through the cell cycle before being treated with 0 or 20 J/m^2^ UV-C. Cells were allowed to recover for 1 hour before chromatin isolation. UV-C damage induced an increase in chromatin-bound RPA2, and RPA2 phosphorylated at S33, an ATR-dependent modification induced by DNA damage (*47*). PrimPol bound to chromatin in G_1_ and S phases, with no observable binding in G_2_ (Fig. 6B and fig. S9B) (*42*). However, 1 hour after 20 J/m^2^ UV-C damage, PrimPol^S538A^ was found to be chromatin associated in G_2_, while PrimPol and PrimPol^S538E^ were not observably recruited (Fig. 6B). We also followed PrimPol’s chromatin dissociation across the cell cycle and found loss of chromatin binding as cells progressed into G_2_, which correlates with S538 phosphorylation changes described earlier (Fig. 5D and fig. S9C). Together, these data indicate that S538 phosphorylation may play an important role in the regulation of PrimPol’s recruitment to chromatin.

In addition, we observed an increase in RPA foci in cells expressing PrimPol^S538A^ under both undamaged and UV-C–treated conditions, suggesting a possible increase in ssDNA (Fig. 6C and fig. S9D). To look more closely at this, we analyzed levels of native CldU incorporated into DNA to look more specifically for ssDNA. We observed a minor increase in CldU signal in cells expressing mutant forms of PrimPol, and this was also seen with damage (Fig. 6D and fig. S9, E and F). However, this difference was decreased when cells were first treated with BI2536 to inhibit PLK1 with cells expressing PrimPol and PrimPol^S538A^ showing little differences (Fig. 6D and fig. S9, E and F).

In addition, we assessed levels of ssDNA gaps during replication, using the S1 fiber assay to detect repriming events. Under asynchronous conditions, we observed little differences between cell lines. However, when cells were synchronized to late S phase, we noted increased fiber shortening after S1 treatment in cells expressing PrimPol^S538A^, suggesting increased repriming (Fig. 6E). To look at the impact of PLK1 phosphorylation on late S phase repriming, we released cells from thymidine into BI2536 to prevent S538 phosphorylation. Cells were allowed to progress into late S phase, at which point we again measured S1 nuclease cutting. We found that all cell lines showed an increase in replication fork length distribution after PLK1 inhibition and that expression of PrimPol now also leads to a significant decrease in fork length after S1 treatment, mimicking PrimPol^S538A^ expression.

### Phosphorylation of S538 is actively regulated in response to fork stalling

As well as cell cycle changes, PLK1 has previously been shown to regulate protein activity via phosphorylation in response to DNA damage (*27*, *28*). We therefore analyzed whether PrimPol may also be regulated in response to damage/fork stalling by PLK1, through its phosphorylation of S538. When cells were allowed to progress synchronously through the cell cycle in the presence of damage, S538 phosphorylation was delayed along with the cell cycle, but phosphorylation was still detected once the bulk of cells entered G_2_ (fig. S10, A and B). This confirmed that S538 phosphorylation is tightly maintained with cell cycle stage and constitutively activated upon completion of S phase.

**Fig. 7.**
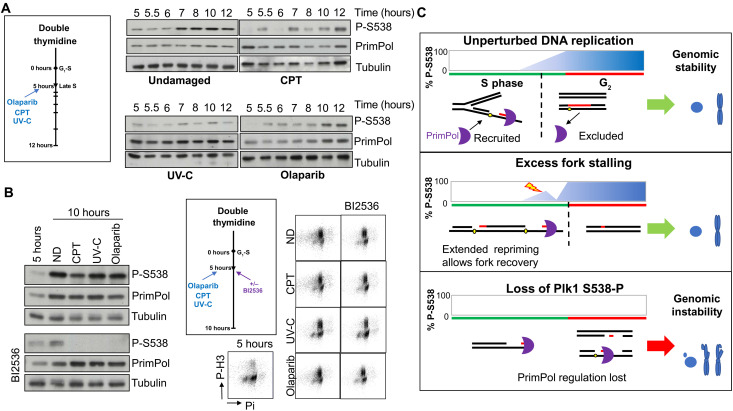
S538 phosphorylation of PrimPol changes in response to DNA damage and cell cycle progression. (**A**) PrimPol S538 phosphorylation levels were followed after damage in late S phase. Cells were released from a double thymidine block into nocodazole containing media for 5 hours before the addition of damage and analyzed by Western blot. (**B**) Loss of PrimPol S538 phosphorylation was monitored, after late S phase damage, in the presence of the PLK1 inhibitor to prevent rephosphorylation. Cells were released from a double thymidine block into media containing nocodazole for 5 hours, before the addition of damage or no damage (ND) in the absence or presence of BI2536 for a further 5 hours. Whole-cell lysates were analyzed by Western blotting and progression into mitosis by P-H3 staining for flow cytometry. (**C**) A schematic model showing the levels of S538 phosphorylation during S-G_2_ and the proposed roles this plays in regulating PrimPol’s usage during the cell cycle and in response to replication stress.

To determine whether active dephosphorylation of S538 was possible in response to damage, we first synchronized cells to the G_1_-S boundary using thymidine and released synchronously for 5 hours to late S phase, the point where S538 phosphorylation begins to appear. Cells were then treated with sufficient doses of UV-C, olaparib, or camptothecin to slow the S phase completion but still ultimately allow cells to progress to mitosis. We found a small but consistent decrease in phosphorylation shortly after damage, compared with unperturbed cells (Fig. 7A and fig. S10C). This was most prominent 1 hour after camptothecin treatment and, in all cases, was resolved around 5 hours after damage induction when most cells were in G_2_ or mitosis. However, flow cytometry-activated cell sorting (FACS) analysis revealed that 5 hours after release, a large proportion of cells had already entered G_2_ and were therefore unlikely to encounter stalled replication forks. This suggested that we were unable to visualize damage-induced dephosphorylation because of increased phosphorylation in G_2_ cells.

To address this, we repeated the experiment with the addition of the PLK1 inhibitor BI2536, preventing further phosphorylation by PLK1. Cells were released from a thymidine block into late S phase as before, but this time, before damage, cells were treated with BI2536 and allowed to progress through the cell cycle for a further 5 hours, at which point most cells had reached mitosis (Fig. 7B). In the absence of BI2536, damaged cells showed a similar increase in phosphorylation to untreated cells (Fig. 7B). In contrast, where PLK1 was inhibited, no increase in phosphorylation was observed upon G_2_-M entry in the absence of damage. Notably, cells treated with olaparib, camptothecin, or UV-C showed a significant loss of S538 phosphorylation (Fig. 7B). This indicates that PrimPol is actively dephosphorylated in response to damage in late S phase. We hypothesize that this dephosphorylation is important for proper utilization of PrimPol in late S phase, where it may only be required after damage due to an excess abundance of stalled forks.

## DISCUSSION

PrimPol-dependent repriming offers many advantages as a mechanism for restarting arrested forks. It enables stalled DNA synthesis to resume by bypassing a diverse range of impediments, without interacting with the obstacle itself, as occurs in TLS. In addition, when PrimPol mediates replication restart, it likely only incorporates a small number of nucleotides before disassociating, due to its low processivity, minimizing any mutagenic events. However, PrimPol-mediated repriming must be tightly regulated, as excessive repriming could lead to increased accumulation of ssDNA gaps that interfere with other key pathways, such as transcription and replication.

In this study, we establish that PrimPol is regulated by PLK1 phosphorylation and that the levels of this modification change throughout the cell cycle (Fig. 5D). Phosphorylation increases as DNA replication is completed, coinciding with a suppression of PrimPol’s recruitment as cells progress into G_2_ (Fig. 6B). Preventing S538 phosphorylation leads to decreased survival after DNA damage or replication stress, an increase in genomic instability in both damaged and unperturbed cells (Figs. 2A and 3, A and B), and deregulated PrimPol recruitment outside of S phase. These phenotypes can be entirely rescued by mutation of the RPA binding domains to prevent chromatin binding (Fig. 2, F and G) and partially rescued by mutations to the ZF domain (Fig. 4, A to D). PLK1-dependent phosphorylation can also be delayed or removed when cells experience replication stress (Fig. 7, A and B). These findings support a model whereby PrimPol usage at stalled replication forks is dynamically regulated by phosphorylation (Fig. 7C). Together, these results highlight the importance of regulating repriming and maintaining a balance between the multiple DDT pathways.

### Regulation of PrimPol by PLK1 alters pathway availability

The role of PLK1 as a highly conserved regulator of mitosis is well established (*48*). Outside of mitosis, PLK1 has been proposed to play roles in S phase, although recent evidence suggests that DNA replication itself suppresses PLK1 activity and its levels do not increase until the bulk of DNA synthesis is complete (*25*, *43*, *48*, *49*). Phosphorylation of PrimPol by PLK1 closely aligns with this profile. PrimPol displays low levels of S538 phosphorylation in early S phase and higher levels in late S-G_2_ (Fig. 5D). Phosphorylation at the end of S phase may potentially operate to keep PrimPol away from replication forks in common fragile sites, which are replicated in late S phase (*50*). Following a similar pattern, PLK1 has also been shown to phosphorylate BRCA2 as cells complete S phase, with phosphorylation levels peaking during mitosis (*26*). As with PrimPol, the cell cycle–dependent phosphorylation of BRCA2 can be suppressed by the application of DNA-damaging agents.

As it has potentially genotoxic priming activity, the cell’s requirements for PrimPol are likely to significantly change throughout the cell cycle. Our data support a model where phosphorylation by PLK1 at the end of S phase negatively regulates PrimPol’s activities as cells enter G_2_. Loss of this regulation leads to inappropriate PrimPol usage outside of S phase. This likely leads to unscheduled repriming events causing increased ssDNA gaps that interfere with G_2_ pathways, as well as at transcription bubbles. Cell cycle–dependent phosphorylation provides an innate regulatory mechanism whereby proteins can be dynamically and reversibly regulated, without the need to be degraded and resynthesized. Our study has demonstrated that although PrimPol is gradually repressed toward the end of S phase, if cells experience significant replication stress in late S phase, then PrimPol can be reactivated by dephosphorylation (Fig. 7C). This implies the usage of one or more phosphatases working in concert with PLK1 to regulate PrimPol, although more work is required to uncover this regulatory mechanism.

### The outcomes of dysregulation of the PrimPol pathway

Cells expressing PrimPol^S538A^, which cannot be phosphorylated, are sensitive to olaparib, camptothecin, UV-C, and cisplatin (Figs. 2A and 3A). These treatments induce replication stress, which leads to fork stalling and the generation of ssDNA. ssDNA is particularly sensitive to damage and hypermutation, due to greater exposure of the bases to oxidative and chemical damage (*51*). Therefore, ssDNA is bound by RPA for protection during replication and repair. Without regulation, PrimPol may be aberrantly recruited to these regions of ssDNA through its interaction with RPA, when alternative mechanisms of DDT may be better suited. As a result, an increase in ssDNA is observed in PrimPol^S538A^ cells (Fig. 6, C to E).

A recent study indicated that the ssDNA gaps left behind by PrimPol-mediated repriming are repaired by Rad51-dependent HR pathways (*52*). To maintain cell survival and suppress DSB formation, ssDNA gaps must be repaired before the cell progresses through the cell cycle, and a marked increase in gaps is likely to require much of the cell’s HR machinery. In addition, excess ssDNA may potentially deplete the cell’s RPA pool, leaving ssDNA exposed and sensitive to further damage, ultimately leading to replication catastrophe (*16*, *53*, *54*). Recent work has also suggested that cancer cell survival depends, in part, on a shift in the balance between DDT pathways, with cancer cells increasingly dependent on TLS polymerases to suppress excessive ssDNA gap formation (*55*). This recent study lends support to a hypothesis that deregulation of PrimPol leads to increased recruitment in late S-G_2_, leading to increased gap formation and decreased cell fitness and genomic stability.

In addition to DNA-damaging agents, loss of PrimPol regulation also causes sensitivity to the PARP inhibitor olaparib. PARP plays multiple roles in the maintenance of genomic stability, including repair of SSBs and unligated Okazaki fragments, as well as promoting replication fork reversal (*33*, *36*, *56*). It has been reported that PARP1 acts in conjunction with CARM1 to promote fork reversal, and its inhibition by olaparib leads to increased utilization of PrimPol (*15*). Our findings also show that replication fork speeds increased after olaparib treatment, although notably, this was not increased further in PrimPol^S538A^ cells. We observed similar sensitives with camptothecin but have shown that all mutants are able to reinitiate replication after stalling in S phase. This suggests that regulation of PrimPol by PLK1-dependent phosphorylation is less important in S phase and mainly acts to regulate PrimPol once the bulk of replication is completed.

### PrimPol^S538A^ phenotypes require RPA binding but are only partially dependent on the ZF

Although we show that phosphorylation of PrimPol regulates its activities throughout the cell cycle, the details of how this is achieved are still to be determined. The location of this PLK1 modification between the RBMs initially implied that S538 phosphorylation may regulate PrimPol’s RPA interaction. However, we observed no difference in the interaction of PrimPol^S538A^ or PrimPol^S538E^ with chromatin or with RPA70 in vivo or in vitro. However, PrimPol’s interactions with RPA at stalled forks may be dependent on other modifications or binding partners that are yet to be uncovered. Genomic instability phenotypes induced by PrimPol^S538A^ expression are, however, dependent on RPA binding and recruitment to chromatin (Fig. 2F).

While PrimPol^ZF^ mutant cannot initiate de novo primer synthesis, it retains polymerase activity and can extend existing primers (*7*, *37*). Most of the PrimPol^S538A^ genotoxicity was lost by mutating the ZF domain. These cells showed increased survival after genotoxic stress and decreased chromosome breaks compared to PrimPol^S538A^ alone. This aligns with observations from other in vivo complementation studies, where it has been found that most phenotypes observed upon PrimPol depletion are not complemented by PrimPol^ZF^ (*4*, *7*, *57*). These data suggest that PrimPol^S538A^ genotoxicity is largely due to PrimPol’s repriming activity. However, cells expressing PrimPol^ZF,S538A^ are still more damage sensitive than PrimPol^ZF^ (Fig. 4). We hypothesize that this is likely to be due to the aberrant recruitment of PrimPol^S538A^ to chromatin, which is maintained after the addition of the ZF mutations (fig. S7F). Aberrant recruitment, without the ability to reprime, may block alternative mechanisms of DDT or repair and delay fork restart, leading to this partial phenotype.

In summary, this study establishes that PLK1-dependent phosphorylation of PrimPol prevents aberrant recruitment and repriming that could otherwise lead to significant genomic instability. Our data highlight the importance of appropriately regulating PrimPol’s recruitment following replication fork stalling and throughout the cell cycle. While this study identifies that PrimPol is specifically regulated by PLK1, it is likely that additional mechanisms also regulate PrimPol and other DDT pathways to ensure that cells respond appropriately in the immediate aftermath of replication stress. The discovery of PLK1’s role in regulating PrimPol’s deployment highlights other important functions that this major cell cycle kinase undertakes outside of mitosis, including acting as key regulator of genome stability.

## MATERIALS AND METHODS

### In vitro kinase assay

To confirm PLK1 phosphorylation of PrimPol, 1 μg of purified PrimPol (WT or S538A) was incubated in a 20-μl reaction with NEB protein kinase buffer, 500 μM ATP, and 20 μg of PLK1 (Merck). The reaction was incubated at 30°C for 2 hours before addition of Laemmli sample buffer and boiling. Samples were then analyzed by Western blot with a total PrimPol and P-S538–specific antibodies.

### Plasmids and mutagenesis

pCDNA5 containing N-terminally Flag-tagged *PrimPol* was used to express PrimPol in HEK293 cells as described previously (*30*). *PrimPol* was cloned into the Sleeping Beauty plasmid pSB*tet* following polymerase chain reaction (PCR) amplification with primers PP SB using a NEBuilder HiFi DNA assembly cloning kit (New England Biolabs) (*31*). A range of mutants were generated by site-directed mutagenesis as described previously (*6*, *7*, *30*); briefly, PCR was carried out with Phusion (New England Biolabs) along with the relevant primers described in table S1, and products were transformed into *Escherichia coli*. Plasmids were purified, and the generation of the desired mutation was confirmed by Sanger sequencing (GATC).

### Human cell culture

HEK293 cells were grown at 37°C, 5% CO_2_ in Dulbecco’s modified Eagle’s medium (DMEM) supplemented with 10% fetal calf serum, 1% l-glutamine, and 1% penicillin/streptomycin. RPE1 cells were grown in DMEM/F12 supplemented with 10% fetal calf serum, 1% l-glutamine, and 1% penicillin/streptomycin.

To generate *PrimPol* knockout cell lines, guide oligos, 1 (5′-TTATCATCCGTATACAGGCCAAGATTGTCCAAGCCAGAAGAACCAC-3′) or 2 (5′-CCATCTATATGGAGGCTGTTTCATCGACAAGCTCAAGCTTTTAATTTTG-3′) targeted to the first exon of *PrimPol*, were cloned into plasmid pSpCas9(BB) as described (*58*). These plasmids were transfected into Flp-In HEK293 T-REx cells, which were separated into single cells in 96-well plates, 3 days after transfection, and grown to form single colonies. Single colonies were selected and expanded, and a proportion were taken for PCR across the target region. PCR was carried out with primer sets KO1, across the guide targeted site, and KO2, far away in exon 7. Clones that showed a change in product size or loss of the target site product, while the exon 7 product was unchanged, were expanded further and tested for loss of PrimPol by Western blotting with a PrimPol-specific antibody (*12*). The genetic change was also confirmed by sequencing of the PCR product generated across the deleted region.

To generate cell lines expressing mutant forms of PrimPol, Flp-In HEK293 T-Rex cells were cotransfected with pOG44 and pCDNA5 containing Flag-tagged *PrimPol* using calcium phosphate (*59*). Cells were selected with hygromycin (100 μg/ml) and blasticidin (15 μg/ml) for approximately 2 weeks, and the resulting resistant clones were pooled. PrimPol was then expressed in these cells by the addition of doxycycline (10 ng/ml). Growth rates of different cell lines were calculated in the presence of doxycycline (10 ng/ml) by counting cells at approximately 24-hour intervals using a hemocytometer, and growth curves were then used to calculate the doubling time using an online tool (*60*).

### PLK1 disruption in vivo

To inhibit PLK1, 10 nM BI2536 (Merck) was added to cell media for 16 hours before protein induction by doxycycline (10 ng/ml). Phosphorylation of protein after BI2536 treatment was assessed by Western blot.

RPE1 PLK1-AS (*42*) cells were cotransfected with 2 μg of pSB*tet*-*PrimPol* and 100 ng of transposase enzyme plasmid pSB-100X by electroporation and selected for 10 days using puromycin (2 μg/ml). Clonal cell lines were generated from single cells and screened for incorporation of BFP (blue fluorescent protein). To inactive PLK1 in these cells, cells were treated with 1 μM 3-MB-PP1, followed by induction of protein expression using doxycycline (100 ng/ml).

### Western blotting and antibodies

To check protein expression, cells were induced by the addition of doxycycline (10 ng/ml) for 24 hours, and 30 μg of total cell lysate was analyzed by Western blotting with a PrimPol antibody in comparison to α-tubulin controls. To look specifically at PrimPol phosphorylation, a phospho-peptide antibody was generated (Eurogentec). Antibodies were raised in rabbits to the peptide ac- ELAEAAEN-S*(PO3H2)*-LLS+C –conh2 and affinity-purified. The specificity of the antibody was confirmed by Western blotting of phosphatase-treated cell lysate. Briefly, cells were lysed in radioimmunoprecipitation assay (RIPA) buffer. Five microliters of PMP buffer (New England Biolabs) and MnCl_2_ (New England Biolabs) was then added to 40 μl of protein sample and then incubated at 30°C for 1 hour with or without 400 U of λ phosphatase. The specificity of the antibody was assessed by Western blot.

### Immunoprecipitation

PrimPol was isolated from HEK293 cells as described previously (*29*). Briefly, approximately 1 × 10^7^ cells expressing Flag-tagged PrimPol were lysed in NETN buffer [150 mM NaCl, 30 mM tris (pH 7.5), 0.5% NP-40, 2.5 mM MgCl_2_, and deoxyribonuclease I (100 μg/ml)] for 30 min at 4°C. Cell lysate was incubated with Anti-Flag M2 magnetic beads (Merck) for 2 hours at 4°C before beads were washed three times with wash buffer [150 mM NaCl, 30 mM tris (pH 7.5), and 0.1% NP-40]. Proteins and interacting partners were eluted with 50 μl of elution buffer [25 mM tris-HCl (pH 7.5), 150 mM NaCl, 1 mM phenylmethylsulfonyl fluoride, and 3xFLAG peptide (200 μg/ml; Merck)] and analyzed against input cell lysate by Western blot.

For mass spectrometry analysis, 10 × 10^7^ cells expressing Flag-tagged PrimPol were lysed in RIPA buffer. Protein was bound by Anti-Flag M2 magnetic beads (Merck) for 2 hours at 4°C before being washed three times in 50 mM ammonium bicarbonate. After overnight on-bead digestion at 37°C by Glu-C protease, the peptides were analyzed on an Orbitrap Exploris 480 [with FAIMS (Field asymmetric Ion mobility spectrometry)] mass spectrometer by the Proteomics Core Facility at CEITEC (Brno, Czech Republic).

### Analysis of *Xenopus* PrimPol C-terminal domain

The *X. laevis* *PrimPol* C-terminal domain (CTD) sequence (amino acids 510 to 675) was gene synthesized (Eurofins) and cloned into the glutathione *S*-transferase expression vector pGEX-KGH. The protein was expressed in BL21 *E. coli* and purified on glutathione agarose (Sigma-Aldrich). Purified protein was dialysed into XB buffer and incubated with *Xenopus* egg extract treated with aphidicolin (100 μg/ml). Sperm pronuclei were added (5 × 10^3^/ml of extract) and incubated at 21°C for 80 min. Extract and nuclei were diluted with XB buffer containing 0.25% Triton X-100, and chromatin was recovered by centrifugation through 30% sucrose. The chromatin pellet was washed extensively with XB buffer and resuspended in XB buffer containing benzonase (2 U/μl). Insoluble material was pelleted by centrifugation, and the soluble supernatant was applied to glutathione agarose beads. Protein was separated by SDS–polyacrylamide gel electrophoresis and in-gel digested with trypsin and chymotrypsin (Promega) overnight at 37°C before analysis by mass spectrometry (LTQ Orbitrap XL/ETD).

### Cell synchronization

To analyze cells within a specific cell cycle stage, cells were synchronized with a double thymidine block. Cells were treated with 4 mM thymidine for 16 hours before being washed three times in phosphate-buffered saline (PBS) and returned to normal media to continue cycling for 8 hours. Cells were then blocked again with 4 mM thymidine for 16 hours, and cells were washed three times in PBS and then used immediately at G_1_/early S phase or allowed to progress through the cell cycle in standard media for 2 hours, S phase, 6 hours, G_2_, or 14 hours, G_1_. To analyze the effects of damage on phosphorylation, cells were released into media containing 1 μM nocodazole to prevent them from progressing through to the next round of the cell cycle. Cells were then treated at relevant time points with 10 μM olaparib, 50 nM camptothecin, or 20 J/m^2^ UV-C, and samples were later collected at relevant time points for chromosome spreads, protein, or FACS analysis. BI2536 (100 nM) was used to examine the effect of PLK1 inhibition.

Where PrimPol expression was required, doxycycline (10 ng/ml) was included in the media throughout. Cells treated in parallel were tested by flow cytometry to confirm synchronization and cell populations.

### Plating efficiency and colony survival assays

Two hundred cells or a serial expansion dependent on expected toxicity were plated with the addition of doxycycline (10 ng/ml) if protein expression was required and allowed to attach for approximately 16 hours. Cells were then left untreated to analyze plating efficiency or treated with increasing doses of UV-C using a G6T5 Germicidal 9” 6W T5 UV-C lamp (General Lamps Ltd.), or relevant concentrations of drugs were added. In the case of cisplatin, drugs were washed off after 6 hours. Colonies were allowed to form for approximately 10 days, and cells were stained with 1% methylene blue for counting. Sensitivity was measured in relation to plating efficiency calculated from undamaged controls.

### Chromatin binding analysis

DNA bound protein populations were analyzed by chromatin assay as described previously (*6*). Approximately 7 × 10^6^ cells were grown in doxycycline (10 ng/ml) for at least 16 hours before being treated with 0 or 20 J/m^2^ UV-C and allowed to recover for 6 hours. Cells were collected, and a quarter were resuspended in 50 μl of NETN buffer [150 mM NaCl, 5 mM EDTA, 50 mM tris (pH 7.5), and 0.5% NP-40]. The remaining cells were incubated in 150 μl of CSK buffer [100 mM NaCl, 300 mM sucrose, 3 mM MgCl_2_, 10 mM Pipes (pH 6.8), 1 mM EGTA, and 0.2% (v/v) Triton X-100] on ice for 5 min before being pelleted at 4°C with the supernatant containing soluble proteins. The pellet containing chromatin-bound proteins was washed twice in PBS and resuspended in Laemmli sample buffer and boiled for 10 min. Proteins were analyzed by Western blotting relative to whole-cell fraction using antibodies.

### RPA foci

Cells were plated on poly-lysine–coated coverslips in doxycycline (10 ng/ml) for at least 16 hours. Cells were either left undamaged or treated with 6 J/m^2^ UV-C and allowed to recover for 24 hours. Cells were preextracted with CSK buffer [100 mM NaCl, 300 mM sucrose, 3 mM MgCl_2_, 10 mM Pipes (pH 6.8), 1 mM EGTA, and 0.2% (v/v) Triton X-100] for 10 min on ice before being fixed with 3% paraformaldehyde. Cells were stained with the relevant antibodies, mouse anti-RPA2 and anti-mouse Alexa Fluor 488, EdU was labeled by click chemistry, and slides were mounted in VECTASHIELD with 4′,6-diamidino-2-phenylindole (DAPI) (Vector Labs) (table S2). Slides were analyzed on an Olympus IX70 fluorescent microscope and analyzed using ImageJ.

### ssDNA staining

Cells were plated on poly-lysine–coated coverslips in doxycycline (10 ng/ml) and 10 μM CldU for 48 hours. Cells were washed before being labeled with 10 μM Edu in the absence or presence of 10 μM olaparib or 50 nM camptothecin for 2 hours. Where PLK1 inhibition was required, cells were treated with 100 nM BI2536 for 1 hour before any damage, and BI2536 was maintained throughout. Cells were preextracted with 2× CSK buffer [100 mM NaCl, 300 mM sucrose, 3 mM MgCl_2_, 10 mM Pipes (pH 6.8), 1 mM EGTA, and 0.4% (v/v) Triton X-100] for 10 min on ice before being fixed with 3% paraformaldehyde. Cells were stained with the relevant antibodies, rat anti-BrdU and anti-rat Alexa Fluor 488, before click chemistry was used to label EdU, and slides were mounted in VECTASHIELD with DAPI (table S2). Slides were analyzed on an Olympus IX70 fluorescent microscope and analyzed using ImageJ.

### Micronuclei assays

To analyze micronuclei, cells were plated in doxycycline (10 ng/ml) for 16 hours before being treated with 0 or 5 J/m^2^ UV-C, 0.5 μM olaparib, or 10 nM camptothecin. Forty-eight hours after treatment, cells were cytospun onto glass slides, fixed with paraformaldehyde, and mounted in VECTASHIELD with DAPI (Vector Labs). Cells were analyzed for the presence of micronuclei on a Nikon E400 fluorescent microscope. To analyze the effect of cell cycle position at the time of damage, cells were plated overnight as before in doxycycline. They were then labeled with 10 μM EdU for 30 min before being treated with 0 or 5 J/m^2^ UV-C. Cells were either collected immediately or allowed to recover for 24 or 48 hours before being cytospun and fixed in paraformaldehyde. EdU incorporation was labeled using the click-it reaction using sulfo-CY5 azide (Jena Biosciences) as described previously (*12*, *61*).

### Chromosome spreads

To analyze the occurrence of chromosome breaks, cells were first grown for 96 hours in the presence of doxycycline (10 ng/ml) alone or 48 hours with 0.5 μM olaparib or 10 nM camptothecin. Nocodazole (1 μM) was added for the final 16 hours before the cells, now largely stalled in mitosis, were collected. Cells were swollen in 75 mM KCl at 37°C before being fixed in 3:1 methanol:acetic acid. Cells were dropped onto glass slides, and after drying, chromosomes were stained with Giemsa (Merck) and mounted in Eukitt Quick-hardening mounting medium (Merck). Slides were analyzed on a Nikon E400 fluorescent microscope.

To look in more detail at chromosomal alterations, SCEs were analyzed as described previously (*62*). Briefly, cells were grown in doxycycline (10 ng/ml) and 10 μM BrdU for 48 hours. Cells were blocked in mitosis and collected, spread, and dried as described above. Chromosomes were then stained with Hoechst (10 μg/ml) before being washed in SSC (150 mM sodium chloride and 15 mM sodium citrate), exposed to UV light for 1 hour, and then incubated in SSC buffer for a further 1 hour at 60°C. Slides were then stained with Giemsa and mounted and viewed as described above.

### Fiber assays

Replication fork speed and stalling were analyzed on DNA fibers as described previously (*6*). Briefly, approximately 10 × 10^4^ cells were incubated in doxycycline (10 ng/ml) for at least 16 hours, and cells were then labeled with 25 μM CldU for 20 min followed by 250 μM IdU for a further 20 min. For fork stalling assays, a pulse of 20 J/m^2^ UV-C was given in between the two labels. Where the effects of damage were analyzed, cells were either first treated with 10 μM olaparib for 2 hours and throughout the labeling or 50 nM camptothecin was added along with IdU label. For analyses of fork restart, cells were labeled as normal with 25 μM CldU for 20 min before the addition of 4 mM HU for 16 hours or 5 μM camptothecin for 1 hour. Drugs were washed off, and cells were released into media containing 250 μM IdU for 60 min. Cells were collected into 150 μl of PBS, and 2.5 μl was lysed directly on slides with 7.5 μl of lysis buffer [20 mM tris (pH 7.5), 50 mM EDTA, and 0.5% SDS]. DNA was spread down the slides using gravity before being fixed with 3:1 methanol:acetic acid. After rehydration, fibers were stained with antibodies to the specific labels, rat anti-BrdU [BU1/75 (ICR1)], mouse anti-BrdU clone B44, anti-rat Alexa Fluor 488, and anti-mouse Alexa Fluor 594 (table S2). Slides were mounted with Fluoromount (Sigma-Aldrich) and imaged on an Olympus IX70 fluorescent microscope and analyzed using OMERO. The S1 fiber assay was adapted from previous protocols (*63*), and cells were labeled as above before being collected and treated with CSK buffer on ice for 10 min. Nuclei were pelleted and treated with 0 or 20 U/ml of S1 (Promega) for 30 min at 37°C before being washed and spread as above.

### Flow cytometry

Cell cycle populations were analyzed using flow cytometry. To confirm synchronization, cells were collected at desired time points and fixed in 70% ethanol at −20°C. To label replicating DNA, cells were treated with 10 μM EdU before collection. EdU-positive cells were then labeled using Click chemistry and sulfo-CY5 azide (Jena Biosciences) (*12*, *61*). Cells were then washed in PBS and labeled with propidium iodide (PI; 5 μg/ml), and RNA was degraded with ribonuclease A (150 μg/ml). To follow progression into mitosis, samples were additionally stained for P-H3. After fixation, cells were permeabilized with 0.2% Triton X-100 in PBS for 10 min before blocking in 3% bovine serum albumin and staining with antibodies to P-H3 followed by anti-rat 488 green (table S2). Cells were then stained for EdU and PI as above. Samples were analyzed using a BD Accuri C6 flow cytometer, and approximately 10,000 cells were quantified using BD CSampler Software. To follow cell cycle progression after damage, cells were first plated in doxycycline (10 ng/ml) for at least 16 hours before being treated with 0 or 5 J/m^2^ UV-C. Cells were allowed to recover for increasing times before being labeled with 10 μM EdU for 30 min and collected as above.

### Purification of recombinant proteins

Full-length human PrimPol and S538A/E mutants were purified as described previously (*7*). Briefly, the proteins were expressed in SHuffle T7 *E. coli* cells (New England Biolabs) overnight at 16°C. Following sonication and isolation by centrifugation, the proteins were purified by affinity chromatography using Ni-NTA affinity resin (Generon), then separated by charge by affinity exchange chromatography on a Hi-Trap Heparin HP column (GE Healthcare), and lastly subjected to size exclusion chromatography on a Superdex 75 gel filtration column (GE Healthcare).

PrimPol CTD (PrimPol_480–560_) and corresponding S538A/E mutants were purified as described previously (*30*). Briefly, the proteins were expressed in BL21 *E. coli* cells overnight at 20°C. Following sonication and isolation by centrifugation, the proteins were purified by affinity chromatography using Ni-NTA affinity resin (Generon), followed by separation by Q-Sepharose (GE Healthcare) and size exclusion chromatography on a Superdex 75 gel filtration column (GE Healthcare).

RPA70N (RPA70_1–120_) was purified as described previously (*30*). Briefly, the protein was expressed in BL21 *E. coli* cells overnight at 20°C. Following sonication and isolation by centrifugation, RPA70N was purified using Ni-NTA affinity resin (Generon) with a gradient elution. The polyhistidine tag was cleaved by thrombin overnight at room temperature, and the product passed through Ni-NTA affinity resin (Generon) to separate the protein from the tag. Last, the protein was separated by a size exclusion chromatography step on a Superdex 75 gel filtration column (GE Healthcare).

### Analytical size exclusion chromatography

Protein interactions were analyzed by analytical size exclusion chromatography as described previously (*29*). Briefly, a Superdex 75 10/300 GL gel filtration column (GE Healthcare) was preequilibriated in a buffer containing 50 mM tris-HCl, 100 mM NaCl, and 2 mM TCEP (Tris(2-carboxyethyl)phosphine hydrochloride). First, individual proteins were loaded at a concentration of 35 μM to provide baseline elution volumes for each. To test protein interactions, the CTD variants were mixed with RPA70N at 35 μM. Interactions were identified by a shift in the chromatograph peaks relative to the respective baseline elution volumes.

### Primase assays

Increasing concentrations of protein (0.5, 1, 2, and 4 μM) were incubated in 20-μl reactions containing 10 μM ssDNA template (Cy5-CCAACCTTTATATTGCCAATCTCTAACCTTTTTCCCATTTACATATAGTddG) with 100 μM deoxynucleotide triphosphates (dNTPs), 2.5 μM FAM-y-GTP, 10 mM bis-tris-propane-HCl (pH 7), 10 mM NaCl, 2 mM MnCl_2_, and 0.5 mM TCEP. The reaction was carried out at 37°C for 30 min and stopped by the addition of 15 μl of stop buffer (60% formamide, 5 mM EDTA, 0.025% SDS, 0.09% xylene cyanol, and 6 M urea). Excess labeled NTP was removed by ethanol precipitation. Primers were resuspended in 20 μl of loading dye (95% formamide with 0.25% bromophenol blue and xylene cyanol). Samples were boiled and resolved on a 20% polyacrylamide/7 M urea/TBE gel at 25 W for 2 hours. Fluorescently labeled primers were detected using a Fujifilm FLA-5100 image reader.

### Polymerase assays

A template oligonucleotide (GACTACTATCTCGACTATATACTATTGCTTCTACGAAGACCTTCA) was annealed to a complementary fluorescently labeled DNA primer (FAM-TGAAGGT-CTTCGTAGAAGC). Protein (50 nM) was incubated with 30 nM annealed primer-template substrate, 10 mM bis-tris-propane-HCl (pH 7), 10 mM MgCl_2_, 10 mM NaCl, 0.5 mM TCEP, and 100 μM dNTPs to a final volume of 20 μl. The reactions were performed at 37°C and stopped at 2, 5, 10, and 15 min by the addition of 20 μl of stop buffer (60% formamide, 5 mM EDTA, 0.025% SDS, and 6 M urea). For fidelity assays, similar conditions were used, except for 20 nM primer-template substrate, 100 nM PrimPol, and 200 μM dNTPs and a 30-min reaction time. Samples were boiled and resolved on a 15% polyacrylamide/7 M urea/TBE gel at 25 W for 1.5 hours. Fluorescently labeled oligonucleotides were detected using a Fujifilm FLA-5100 image reader.

### Data analyses

Charts show independent experiments with error bars showing SD, and colony survivals represent three or more independent experiments. Significance was determined using Student’s *t* test or Mann-Whitney for fibers and foci, *P ≤ 0.05, **P ≤ 0.01, ***P ≤ 0.001, and ****P ≤ 0.0001. For fiber experiments, the black line represents the mean of the data where approximately 300 fibers were measured across three independent experiments unless stated otherwise. For cell analysis, approximately 500 cells were counted per independent experiment, and for chromosome spreads, this was 100 or 300 for SCEs. Data analysis was carried out using GraphPad, and images were quantified with ImageJ and OMERO.

## Notes

View/request a protocol for this paper from *Bio-protocol*.
