# Supplementary material for: PLK1 regulates the PrimPol damage tolerance pathway during the cell cycle
Source: Sci Adv. 2021 Dec 3;7(49):eabh1004. doi: 10.1126/sciadv.abh1004 (PMC8641930; doi:10.1126/sciadv.abh1004)
Supplement: Supplementary file 1 — Figs. S1 to S10 Tables S1 and S2 [file sciadv.abh1004_sm.pdf]

Science Advances
AAAS

Supplementary Materials for

PLK1 regulates the PrimPol damage tolerance pathway during the cell cycle

Laura J. Bailey, Rebecca Teague, Peter Kolesar, Lewis J. Bainbridge, Howard D. Lindsay, Aidan J. Doherty*

*Corresponding author. Email: ajd21@sussex.ac.uk

Published 3 December 2021, *Sci. Adv.* **7**, eabh1004 (2021)
DOI: 10.1126/sciadv.abh1004

This PDF file includes:

Figs. S1 to S10
Tables S1 and S2

Figure S1

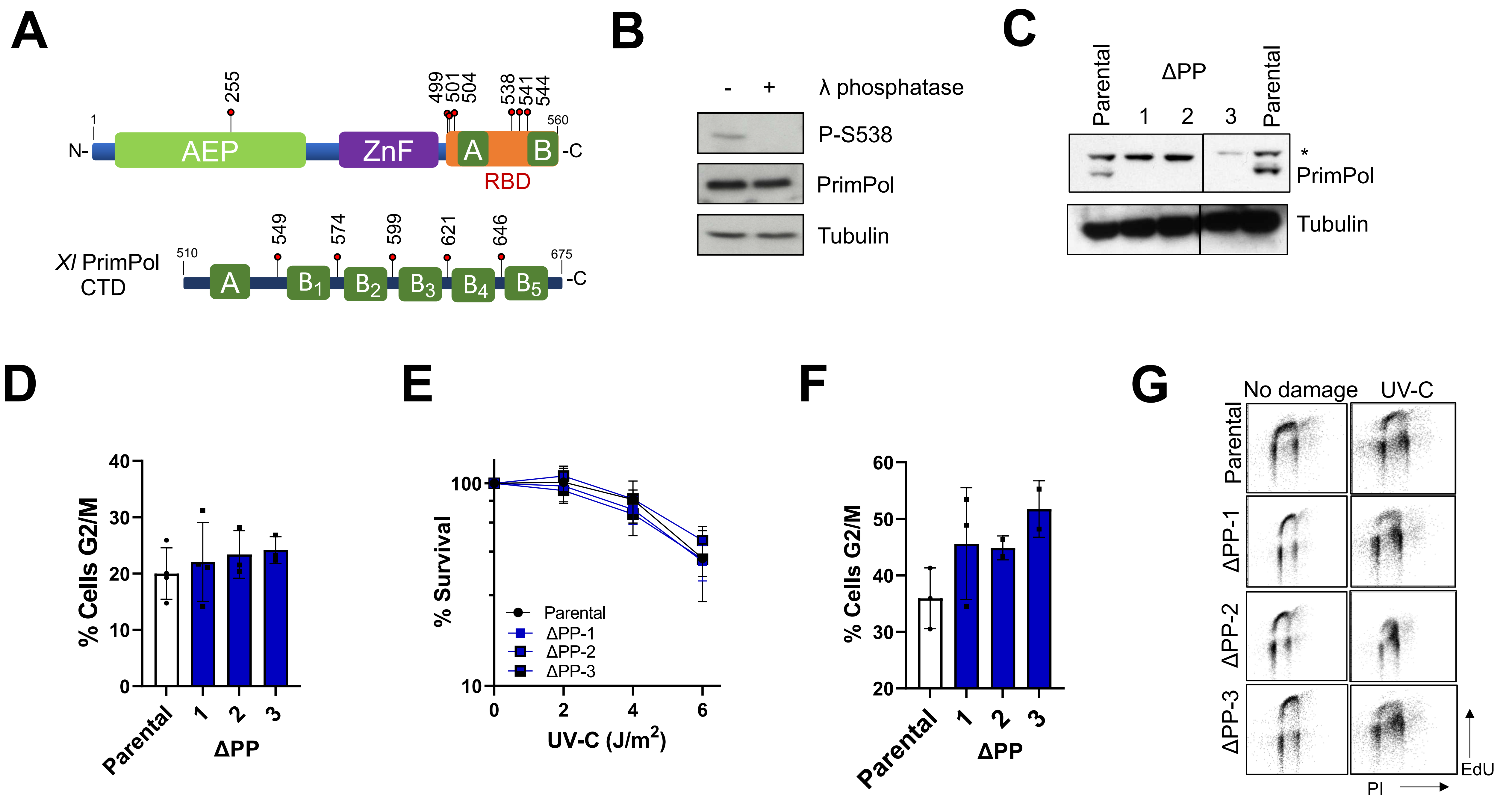

A
255
499 501 504
538 541 544
1
560
N-
AEP
ZnF
A
B
-C
RBD
Xl PrimPol CTD
510
549
574
599
621
646
675
A
B1
B2
B3
B4
B5
-C
B
- +
λ phosphatase
P-S538
PrimPol
Tubulin
C
Parental
ΔPP
1
2
3
Parental
*
PrimPol
Tubulin
D
% Cells G2/M
40
30
20
10
0
Parental
1
2
3
ΔPP
E
% Survival
100
10
Parental
ΔPP-1
ΔPP-2
ΔPP-3
0
2
4
6
UV-C (J/m2)
F
% Cells G2/M
60
50
40
30
20
Parental
1
2
3
ΔPP
G
No damage
UV-C
Parental
ΔPP-1
ΔPP-2
ΔPP-3
EdU
PI

Supplementary Figure 1. Generation and analysis of PrimPol knockout HEK293 cells, ΔPP1-3, and a P-S538 antibody.

(**A**) Phosphorylation sites identified on human and the C-terminal domain (CTD) of *Xenopus laevis* PrimPol by mass spectrometry. (**B**) The specificity of the P-S538 peptide antibody was tested using western blotting of whole cell lysates treated or untreated with λ protein phosphatase. (**C**) Loss of PrimPol protein from individual clones was confirmed by western blotting of whole cell lysate using a total PrimPol antibody in relation to a tubulin total protein marker, * indicates a non-specific band. (**D**) Cell cycle populations were analysed by flow cytometry of PI and EdU labelled cells and figure shows proportion of cells in G2 in an asynchronous population. (**E**) UV-C sensitivity was measured in different clones by colony survival in comparison with parental HEK293 cells. (**F**) Changes in recovery time after UV-C damage was analysed in parental and ΔPP cell lines by flow cytometry. G2 populations were quantified 24 hrs after 0 or 5 J/m^2 UV-C. Representative images shown and quantified in (**G)**.

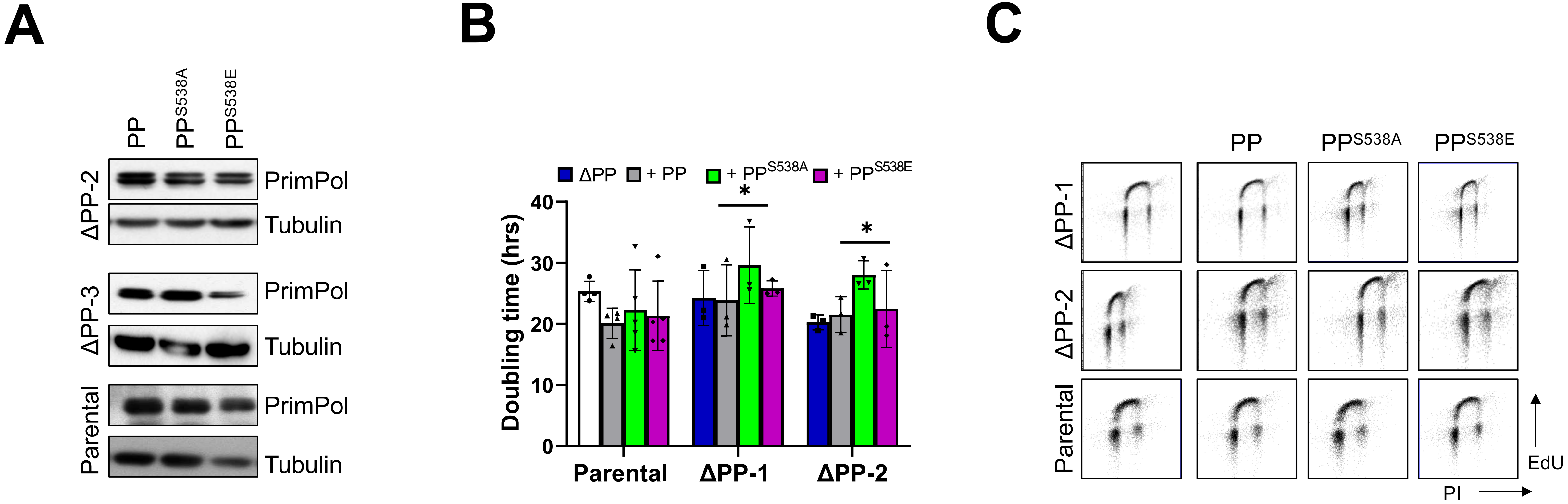
A
PP
PPS538A
PPS538E
ΔPP-2
PrimPol
Tubulin
ΔPP-3
PrimPol
Tubulin
Parental
PrimPol
Tubulin
B
ΔPP
+ PP
+ PPS538A
+ PPS538E
Doubling time (hrs)
40
30
20
10
0
*
*
Parental
ΔPP-1
ΔPP-2
C
PP
PPS538A
PPS538E
ΔPP-1
ΔPP-2
Parental
EdU
PI

Supplementary Figure 2. Expression of PrimPol in HEK293 ΔPP cells

Flag-tagged PrimPol was stably integrated into the HEK293 system using a doxycycline promoter with either no changes or S538A or S538E mutations. Expression was induced with 10 ng/ml doxycycline for 24 hrs and expression was compared in parental HEK293 and ΔPP cell lines by western blotting. (**A**) Growth rates of cell lines expressing different PrimPol constructs was analysed by counting cell numbers every 24 hrs to generate growth curves. (**B**) Figure shows average doubling times for n = 3 independent experiments. (**C)** Representative images from flow cytometry analyses to assess cell cycle in cells expressing different forms of PrimPol.

Figure S3

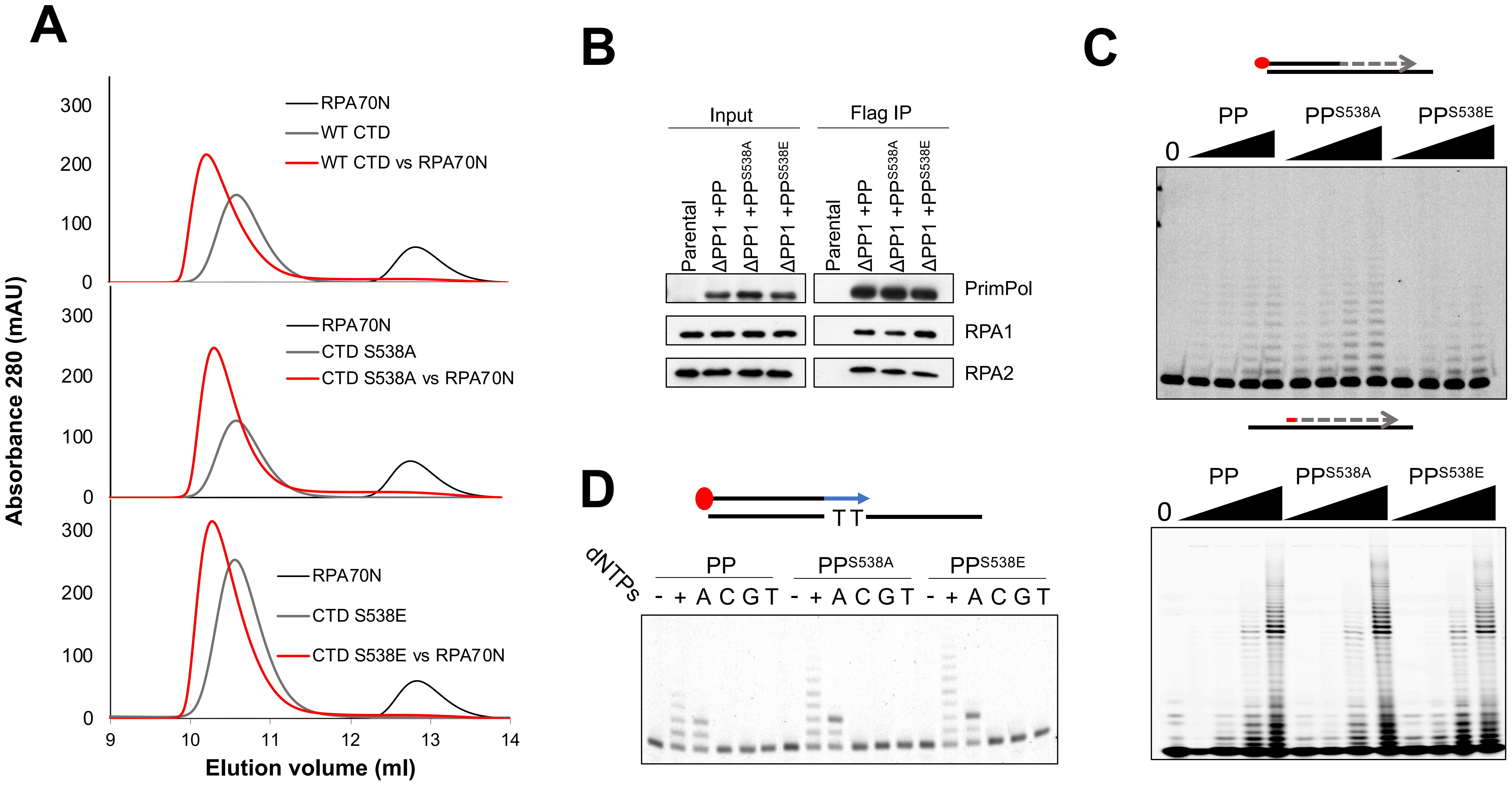
A
RPA70N
WT CTD
WT CTD vs RPA70N
RPA70N
CTD S538A
CTD S538A vs RPA70N
RPA70N
CTD S538E
CTD S538E vs RPA70N
Absorbance 280 (mAU)
Elution volume (ml)
B
Input
Flag IP
Parental
ΔPP1 +PP
ΔPP1 +PPS538A
ΔPP1 +PPS538E
PrimPol
RPA1
RPA2
C
PP
PPS538A
PPS538E
0
D
TT
dNTPs
- + A C G T

Supplementary Figure 3. PrimPol^{S538A} mutation does not affect RPA interaction or primase/polymerase activity.

(**A**) WT, S538A and S538E mutated PrimPol CTD were purified from *E.coli* and their interactions with RPA70N assessed by analytical gel filtration. (**B**) Proteins were immunoprecipitated from doxycycline induced ΔPP HEK293 cells, expressing Flag-tagged PrimPol or parental with only endogenous protein, using Flag magnetic beads and eluted proteins were analysed alongside input material by western blotting using antibodies specific to PrimPol and RPA. (**C**) PrimPol, PrimPol^{S538A} and PrimPol^{S538E} were purified from *E.coli* and polymerase (top panel) and primase (bottom panel) activities of the different proteins compared using fluorescently labelled primers or dNTPs. (**D**) Fidelity of PrimPol incorporation was analysed by looking at utilisation different dNTPs in polymerase extension of a DNA primer opposite TT nucleotides.

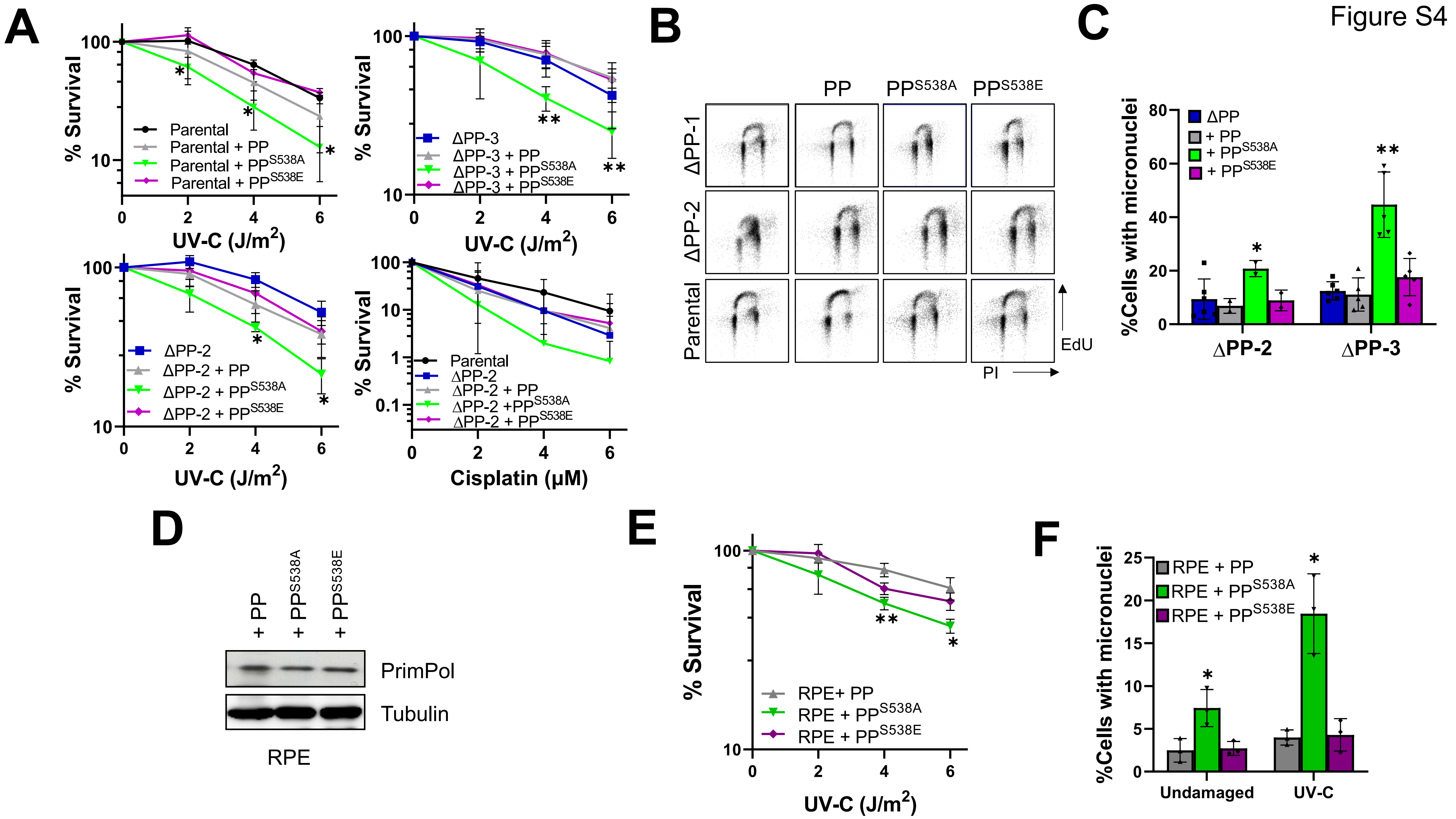
A
% Survival
UV-C (J/m2)
Parental
Parental + PP
Parental + PPS538A
Parental + PPS538E
ΔPP-3
ΔPP-3 + PP
ΔPP-3 + PPS538A
ΔPP-3 + PPS538E
ΔPP-2
ΔPP-2 + PP
ΔPP-2 + PPS538A
ΔPP-2 + PPS538E
Cisplatin (μM)
B
PP
PPS538A
PPS538E
ΔPP-1
ΔPP-2
Parental
EdU
PI
C
%Cells with micronuclei
ΔPP
+ PP
+ PPS538A
+ PPS538E
ΔPP-2
ΔPP-3
D
+ PP
+ PPS538A
+ PPS538E
PrimPol
Tubulin
RPE
E
% Survival
RPE+ PP
RPE + PPS538A
RPE + PPS538E
UV-C (J/m2)
F
%Cells with micronuclei
RPE + PP
RPE + PPS538A
RPE + PPS538E
Undamaged
UV-C

Supplementary Figure 4. Expression of PrimPol^{S538A} effects cell survival and genomic stability.

(**A**) Cell survival was compared in additional cell lines expressing different PrimPol constructs in response to UV-C and cisplatin. (**B**) Representative data showing flow cytometry analysis of cells labelled with EdU and PI 24 hrs after 0 or 5 J/m^2 UV-C treatment, quantification shown in Fig. 2B. (**C**) Percentage of cells with 1 or more micronuclei, 48 hrs after 5 J/m^2 UV-C treatment, compared in different ΔPP clones expressing different PrimPol proteins. (**D**) Analysis of whole cell lysate from RPE cells showing expression of PrimPol by western blot. (**E**) Colony survival after UV-C damage in RPE cells expressing WT or mutant PrimPol. (**F**) After expression of WT or mutant PrimPols, percentage of cells containing micronuclei 48 hrs after treatment with 0 or 6 J/m^2 UV-C was analysed by DAPI staining and microscopy.

Figure S5

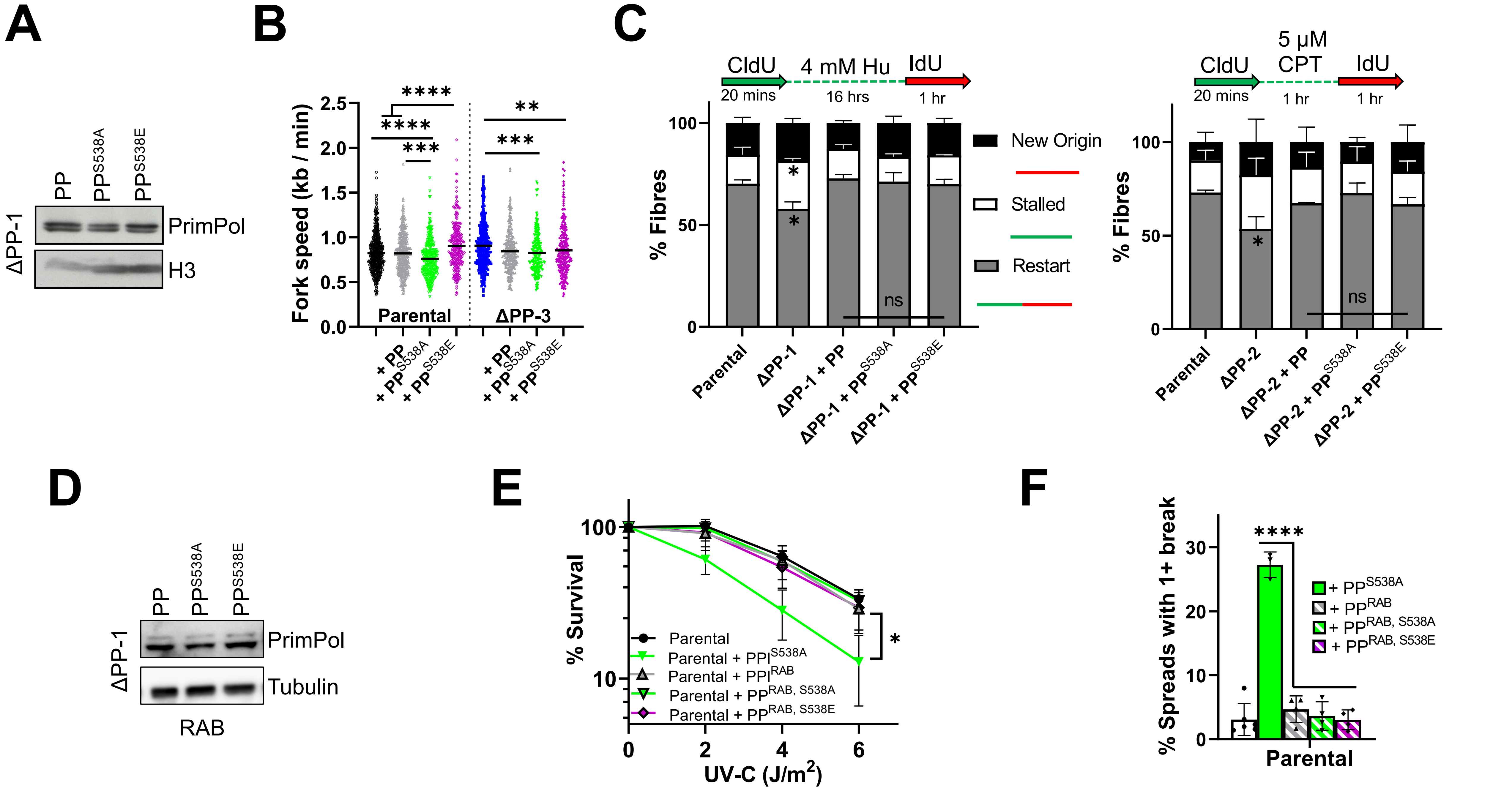
A
B
C
D
E
F
ΔPP-1
PP
PPS538A
PPS538E
PrimPol
H3
Fork speed (kb / min)
****
****
***
**
***
Parental
ΔPP-3
+ PP
+ PPS538A
+ PPS538E
CldU
20 mins
4 mM Hu
16 hrs
IdU
1 hr
% Fibres
New Origin
Stalled
Restart
ns
Parental
ΔPP-1
ΔPP-1 + PP
ΔPP-1 + PPS538A
ΔPP-1 + PPS538E
5 μM CPT
ΔPP-2
ΔPP-2 + PP
ΔPP-2 + PPS538A
ΔPP-2 + PPS538E
Tubulin
RAB
% Survival
UV-C (J/m2)
Parental
Parental + PPIS538A
Parental + PPIRAB
Parental + PPRAB, S538A
Parental + PPRAB, S538E
% Spreads with 1+ break
+ PPS538A
+ PPRAB
+ PPRAB, S538A
+ PPRAB, S538E

Supplementary Figure 5. PrimPol S538A mutation does not affect chromatin binding or fork restart and its toxicity is abolished by loss of RPA binding sites.

(**A**) Cells were treated with 20 J/m^2 and allowed to recover for 6 hrs before chromatin bound proteins were isolated and analysed by western blot. (**B**) Replication fork speed was analysed in additional ΔPP clones and parental cells. Forks were labelled with CldU and IdU for 20 mins each before being spread and labelled. Chart shows data from at least 300 fibres over 3 or more independent experiments, red line represents mean of data. (**C**) Fork restart after HU or CPT stalling was analysed by fibre analysis measuring the number of green only, stalled fibres, restarted, red and green or new origins, red only fibres. (**D**) Flag-tagged PrimPol carrying the RAB mutations alone or in combination with S538A or S538E were stably transfected into parental or ΔPP cells under a doxycycline inducible promoter. Protein was expressed for 24 hrs with 10 ng/ml doxycycline and whole cell extracts were analysed by western blot. (**E**) Parental cells were analysed for UV-C sensitivity when expressing PrimPol^{RAB} mutants by colony survival and the occurrence of chromosome breaks in the absence of damage (**F**).

Figure S6

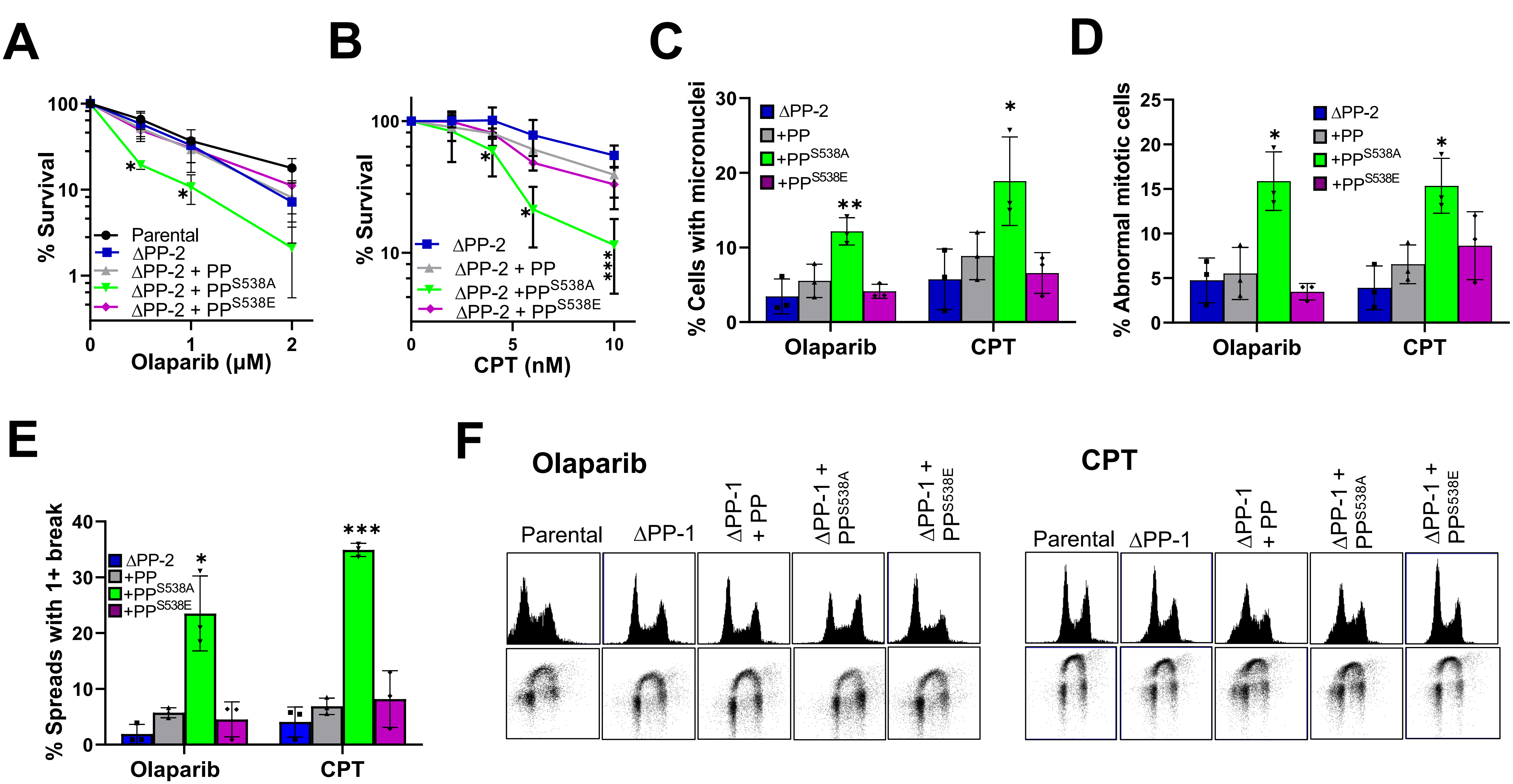
A
100
10
1
% Survival
0
1
2
Olaparib (μM)
Parental
ΔPP-2
ΔPP-2 + PP
ΔPP-2 + PP^S538A
ΔPP-2 + PP^S538E
B
100
10
% Survival
0
5
10
CPT (nM)
ΔPP-2
ΔPP-2 + PP
ΔPP-2 +PP^S538A
ΔPP-2 + PP^S538E
C
% Cells with micronuclei
ΔPP-2
+PP
+PP^S538A
+PP^S538E
Olaparib
CPT
D
% Abnormal mitotic cells
ΔPP-2
+PP
+PP^S538A
+PP^S538E
Olaparib
CPT
E
% Spreads with 1+ break
ΔPP-2
+PP
+PP^S538A
+PP^S538E
Olaparib
CPT
F
Olaparib
Parental
ΔPP-1
ΔPP-1 + PP
ΔPP-1 + PP^S538A
ΔPP-1 + PP^S538E
CPT
Parental
ΔPP-1
ΔPP-1 + PP
ΔPP-1 + PP^S538A
ΔPP-1 + PP^S538E

Supplementary Figure 6. PrimPol S538A mutation sensitises cells to olaparib and camptothecin.

Cell survival after olaparib (**A**) or camptothecin (**B**) was analysed in ΔPP-2 cells expressing different forms of PrimPol. Presence of micronuclei (**C)**, abnormal mitotic cells (**D**), and chromosome breaks (**E**)**,** 48 hrs after olaparib or camptothecin treatment was analysed in a second ΔPP cell line. (**F**) Examples of EdU, PI FACs profiles for cells treated with 2 μM olaparib or 10 nM camptothecin for 48 hrs and quantified in Fig. 3E.

Figure S7

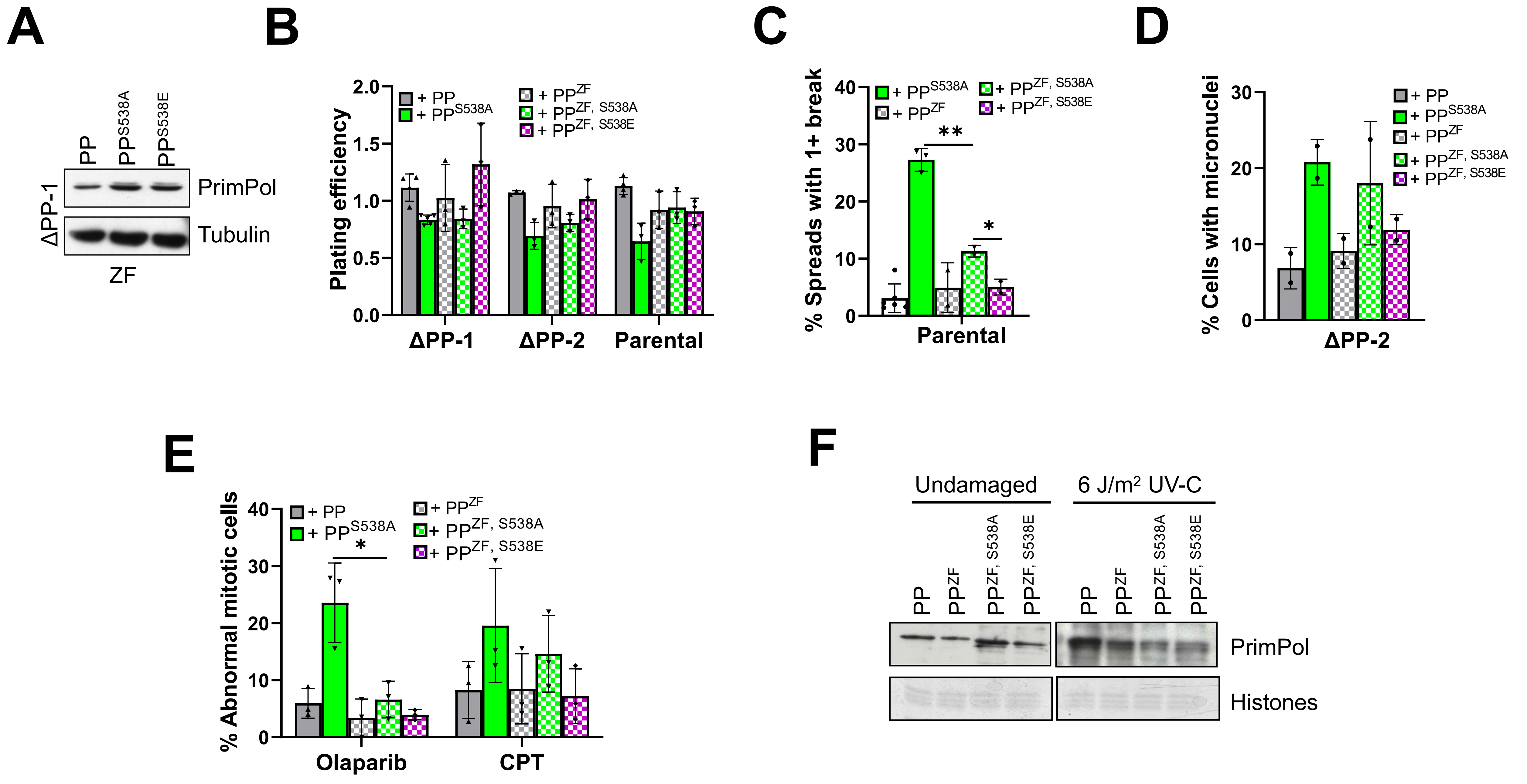
A
B
C
D
E
F
PP
PPS538A
PPS538E
ΔPP-1
ZF
PrimPol
Tubulin
Plating efficiency
2.0
1.5
1.0
0.5
0.0
+ PP
+ PPS538A
+ PPZF
+ PPZF, S538A
+ PPZF, S538E
ΔPP-1
ΔPP-2
Parental
% Spreads with 1+ break
40
30
20
10
0
**
*
% Cells with micronuclei
% Abnormal mitotic cells
Olaparib
CPT
Undamaged
6 J/m² UV-C
PPZF
PPZF, S538A
PPZF, S538E
Histones

Supplementary Figure 7. Effects of ZF mutation on PrimPol^{S538A} toxicity.

(**A**) Flag-tagged PrimPol carrying C419A, H426A, ZF**,** mutations alone or in combination with S538A or S538E were stably transfected into Parental or ΔPP cells under a doxycycline inducible promoter. Protein was expressed for 24 hrs with 10 ng/ml doxycycline and whole cell extracts were analysed by western blot. (**B**) Plating efficiency was used to measure the effects of over expressing different forms of PrimPol in different cell clones. (**C**) Cells expressing PrimPol^{ZF} mutants were grown for 96 hrs before spreading and number of cells with 1 or more chromosome breaks were counted. Data is shown beside that shown previously for cells expressing WT PrimPol mutants. (**D**) Cells expressing PrimPol^{ZF} variants were analysed for percentage with 1 or more micronuclei 48 hrs after 5 J/m^2 UV-C in relation to WT PrimPol mutants shown previously. (**E**) Abnormal mitotic cells were analysed 48 hrs after the addition of camptothecin or olaparib in cells expressing PrimPol^{Zn} in comparison to WT PrimPol. (**F**) Cells expressing PrimPol were treated with 0 or 20 J/m^2 UV-C and allowed to recover for 6 hrs before isolation of chromatin bound proteins and analysis by western blotting.

Figure S8

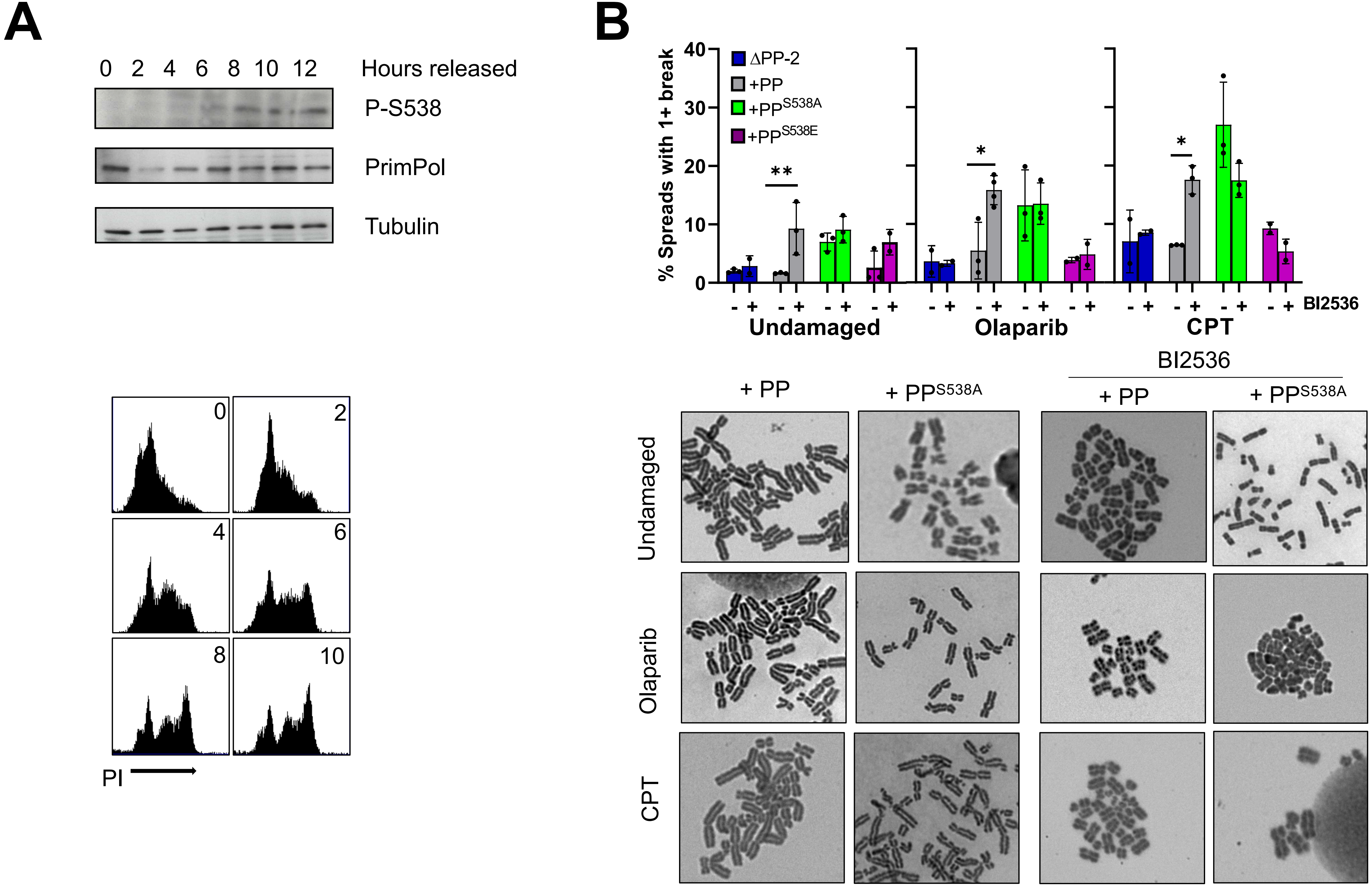

A
0 2 4 6 8 10 12 Hours released
P-S538
PrimPol
Tubulin
0
2
4
6
8
10
PI
B
% Spreads with 1+ break
ΔPP-2
+PP
+PP S538A
+PP S538E
**
*
*
BI2536
Undamaged
Olaparib
CPT
BI2536
+ PP
+ PP S538A
+ PP
+ PP S538A
Undamaged
Olaparib
CPT

Supplementary Figure 8. PrimPol phosphorylation is cell cycle regulated and becomes toxic in the absence of PLK1.

(**A**) RPE cells expressing PrimPol were synchronised by double thymidine block before being released into nocodazole. Cell cycle progression was monitored by facs, lower panel, and the same samples were analysed by western blot for changes in 538 phosphorylation and total PrimPol, alongside tubulin. (**B**) PLK1-dependent toxicity was confirmed in a second clone. Cells were released from a double thymidine block into media containing nocodazole with or without PLK1 inhibitor and camptothecin, olaparib, UV-C or undamaged. After 10 hrs mitotic cells were collected and analysed for chromosome breaks, representative images shown below.

Figure S9

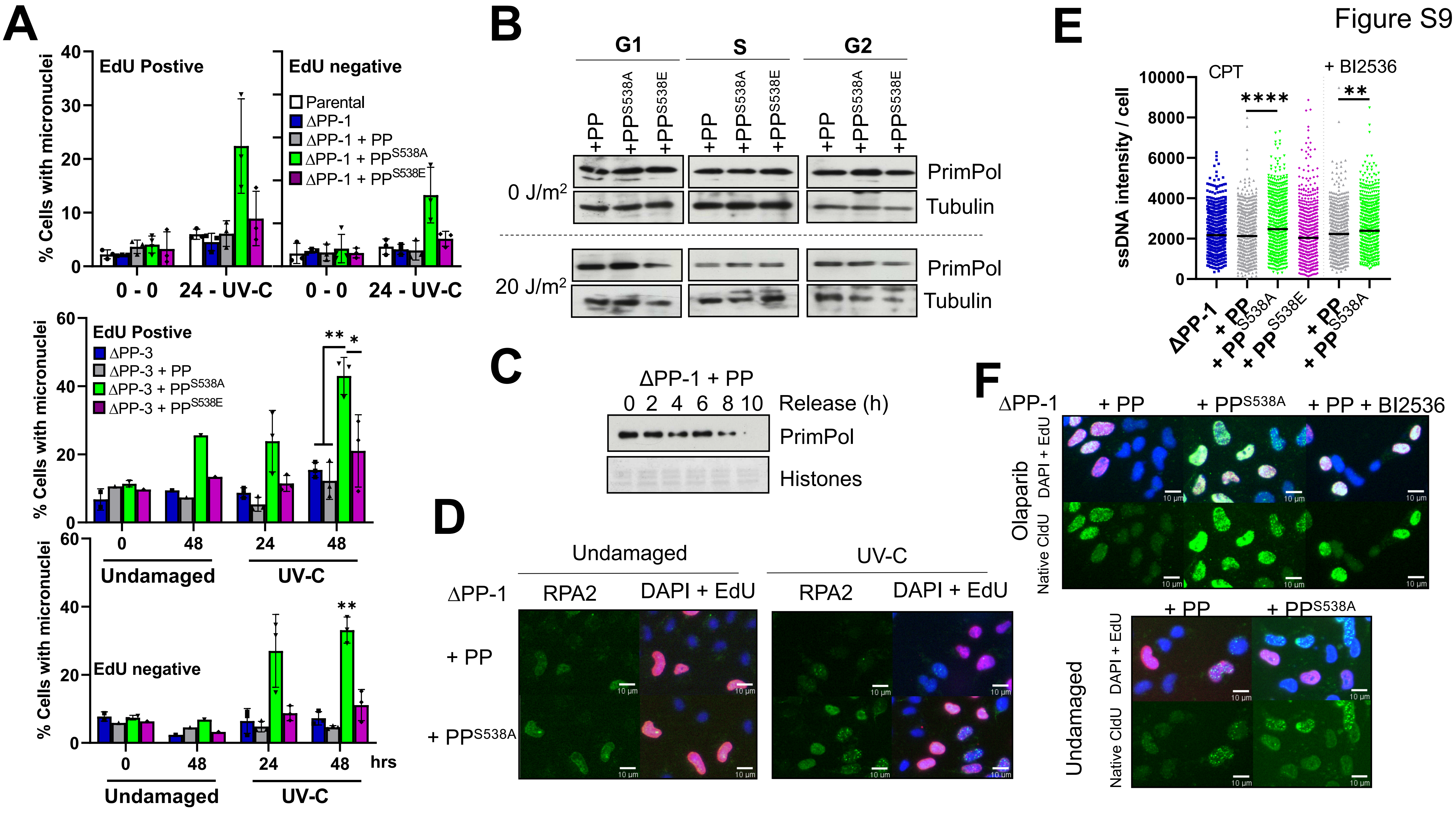
A
EdU Postive
EdU negative
Parental
ΔPP-1
ΔPP-1 + PP
ΔPP-1 + PPS538A
ΔPP-1 + PPS538E
% Cells with micronuclei
0 - 0
24 - UV-C
ΔPP-3
ΔPP-3 + PP
ΔPP-3 + PPS538A
ΔPP-3 + PPS538E
Undamaged
UV-C
hrs
B
G1
S
G2
+PP
+PPS538A
+PPS538E
0 J/m2
20 J/m2
PrimPol
Tubulin
C
ΔPP-1 + PP
0 2 4 6 8 10
Release (h)
PrimPol
Histones
D
Undamaged
UV-C
ΔPP-1
RPA2
DAPI + EdU
+ PP
+ PPS538A
E
CPT
+ BI2536
ssDNA intensity / cell
ΔPP-1
+ PP
+ PPS538A
+ PPS538E
F
ΔPP-1
+ PP
+ PPS538A
+ PP + BI2536
Olaparib
DAPI + EdU
Native CldU
Undamaged
10 µm

Supplementary Figure 9. Unphosphorylated PrimPol is toxic outside of S-phase and leads to increased ssDNA. (**A**) To analyse the effect of UV-C on different cell cycle stages, ΔPP-1 or ΔPP-3 cells were labelled with EdU followed by 0 or 5 J/m^2 UV-C. Cells either, EdU positive or negative, were analysed for the presence of 1 or more micronuclei, immediately after damage, 24 or 48 hrs later. (**B**) whole cell blots from the chromatin samples shown in Fig. 6B. (**C**) Chromatin association of PrimPol was monitored across the cell cycle. ΔPP-1 cells expressing PrimPol were synchronised by double thymidine block and were released to progress through the cell, samples were collected and chromatin associated PrimPol was analysed by western blot alongside histones. (**D**) Representative images showing RPA foci, quantified in Fig. 6C. (**E**) Quantification of native ssDNA signal after 2 hrs treatment with 50 nM camptothecin with or without BI2536. (**F**) Representative images of native CldU staining for ssDNA, quantified in Fig. 6D.

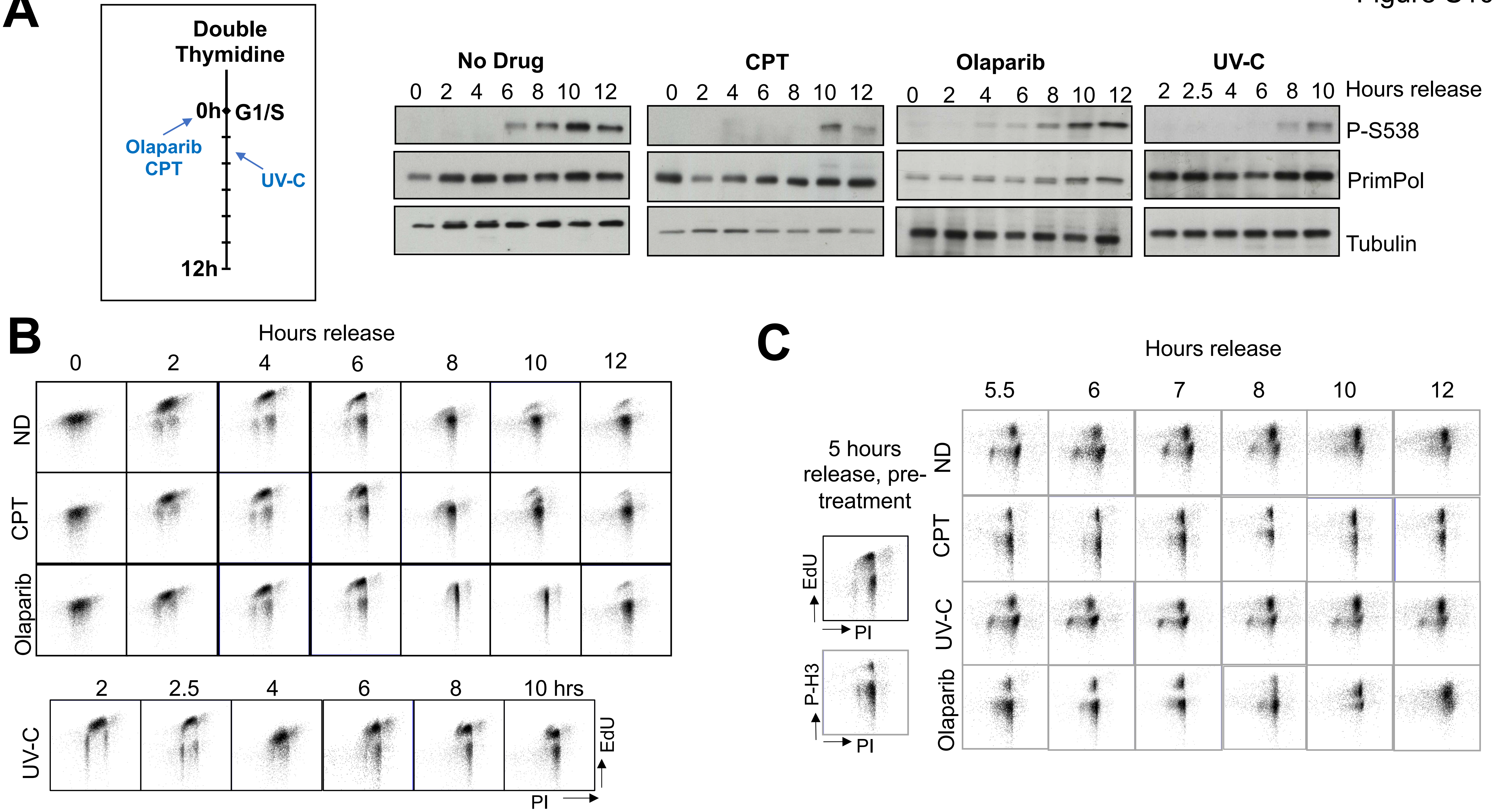
A
Double Thymidine
0h
G1/S
Olaparib
CPT
UV-C
12h
No Drug
CPT
Olaparib
UV-C
0 2 4 6 8 10 12
0 2 4 6 8 10 12
0 2 4 6 8 10 12
2 2.5 4 6 8 10
Hours release
P-S538
PrimPol
Tubulin
B
Hours release
0 2 4 6 8 10 12
ND
CPT
Olaparib
2 2.5 4 6 8 10 hrs
UV-C
EdU
PI
C
Hours release
5.5 6 7 8 10 12
5 hours release, pre-treatment
EdU
PI
P-H3
PI
ND
CPT
UV-C
Olaparib

Supplementary Figure 10. PrimPol S538 phosphorylation is tightly linked with cell cycle procession.

(**A**) Cells were released from a double thymidine block into media containing nocodazole and either immediately treated with olaparib or camptothecin or UV-C irradiated 2 hrs later. Samples were collected over the following 12 hrs and whole cell lysate was analysed by western blot to assess changes in phosphorylation levels. (**B**) Cells were also labelled with Edu and analysed by flow cytometry follow the progression through the cell cycle. (**C**) P-H3 labelling and flow cytometry were used to follow entry of cells into mitosis in synchronised cells where damage was administered at 5 hrs, western blot Fig. 7A.

Table S1 : DNA primers used in this study

**Name**	**Sequence (5'-3')**
S538A fwd	CAGAGAACGCTCTTCTCAGTTATAACAGTGAAGTG
S538A rev	CTGAGAAGAGCGTTCTCTGCAGCTTCAGC
S538E fwd	CAGAGAACGAGCTTCTCAGTTATAACAGTGAAGTG
S538E rev	CTGAGAAGCTCGTTCTCTGCAGCTTCAGC
ZN fwd	GTAAATATCGGTGGGCTGAAAACATTGGAAGAGCCGCTAAGAGTAAT AATATAATG
Zn rev	CTCTTAGCGGCTCTTCCAATGTTTTCAGCCCACCGATATTTACAAATA TCATAAACCAGTAATTC
RA fwd	GGCATTGATCGTGCTTATGCTTTAGAAGCTACTGAAGATGC
RA rev	GCTTCTAAAGCATAAGCACGATCAATGCCATTATCCCAGAC
RB fwd	GAAATTCCTCGTGAACTAGCTATAGAAGTATTACAAGAG
RB rev	CTTCTATAGCTAGTTCACGAGGAATTTCATCCACTTCAC
KO1 fwd	ATGAATAGAAAATGGGAAGCAAAACTG
KO1 rev	GCTTGTCGATGAAATAGTCTCCAGATG
KO2 fwd	GCTTGGCAGTGAAGATGATGATAGC
KO2 rev	GCTTCTCTCCCATGTTATTCTTCACAAC
PP SB fwd	GAAAGGCCTCTGAGGCCACCATGAATAGAAAATGGGAAGC
PP SB rev	ATCTTATCATGTCTATCGATCTACTCTTGTAATACTTCTATA ATTAGTTC

Table S2: Antibodies used in this study

Antibody	Supplier
Rabbit anti - PrimPol	Bianchi *et al. Mol. Cell* **52**, 566-573 (2013)
Rabbit anti - PrimPol	Antibody Genie (#PACO0022224-100)
Rabbit anti - PrimPol P-S538	Eurogentec- this publication
Mouse anti - tubulin	Merck (T5168)
Rabbit anti - H3	Abcam (ab1791)
Rat anti - P-H3 (HTA28)	Abcam (ab10543)
Rat anti-BrdU [BU1/75 (ICR1)]	Abcam (ab6326)
Mouse anti-BrdU Clone B44	BD (347580)
Mouse anti – RPA1	Calbiochem (Na 13)
Mouse anti - RPA2	Calbiochem (Na 18)
Rabbit anti-P(S33)RPA2	Novus Biologicals (NB100-544)
Mouse anti-Cyclin A2	Abcam (ab16720)
Rabbit anti-P(S46)TCTP	Cell Signalling #5251
Anti - rabbit HRP	Abcam (ab6721)
Anti - mouse HRP	Abcam (ab6728)
anti-mouse Alexa Fluor 594	Invitrogen Molecular Probes (A31624)
anti-mouse Alexa Fluor 488	Invitrogen Molecular Probes (A31620)
anti-rat Alexa Fluor 488	Invitrogen Molecular Probes (A21208)
